# High-fat diet impairs microbial metabolite production and aggravates influenza A infection

**DOI:** 10.1186/s12964-025-02367-w

**Published:** 2025-07-31

**Authors:** Franziska Hornung, Harini K. SureshKumar, Laura Klement, Yasmina Reisser, Christoph Wernike, Vivien Nischang, Paul M. Jordan, Oliver Werz, Carsten Hoffmann, Bettina Löffler, Stefanie Deinhardt-Emmer

**Affiliations:** 1 Institute of Medical Microbiology, Jena University Hospital, Am Klinikum 1, Jena, Germany; 2 Institute of Molecular Cell biology, CMB– Center for Molecular Biomedicine, Jena University Hospital, Friedrich Schiller University Jena, Jena, Germany; 3 Department of Pharmaceutical/Medicinal Chemistry, Institute of Pharmacy, Friedrich Schiller University Jena, Jena, Germany

**Keywords:** Microbial metabolites, High-fat diet, Short-chain fatty acids, Gut-lung-axis, Acetate, Influenza A virus, FFAR2, Interferon response

## Abstract

**Background:**

Alterations in the gut microbiom can significantly impact various regions in the human body, including the pulmonary tract. This study investigates alterations in the gut microbiome during a high-fat diet (HFD), particularly short-chain fatty acids (SCFAs), and how these metabolites affect lung infection caused by Influenza A virus (IAV).

**Methods:**

We used a HFD-mouse model to evaluate gut microbiota composition, SCFA levels, and pulmonary outcomes following IAV infection. Microbial changes were analyzed via taxonomic and functional profiling and SCFA levels were measured from non-obese and obese serum donors. Ultimately, acetate’s effects were tested ex vivo in human precision-cut lung slices (PCLS) and in vitro in pulmonary epithelial cells. Mechanistic studies investigated the involvement of the SCFA receptor free fatty acid receptor 2 (FFAR2) and intracellular antiviral pathways.

**Results:**

Our data indicates an increased *Firmicutes/Bacteroidetes* ratio of the gut microbiome and an altered carbohydrate metabolism, leading to reduced SCFA production. Infected HFD mice showed increased IAV titers and sustained microbial alterations. Interestingly, acetate demonstrated antiviral effects in both the human PCLS model and pulmonary cells with an reduced viral replication. These effects depended on FFAR2, which also acts as an IAV co-receptor, as acetate treatment led to FFAR2 internalization and influenced host cell metabolism in our in vitro data.

**Conclusion:**

HFD alters the SCFA production, reducing acetate levels in the gut microbiome. This reduction may lead to higher viral loads and worsened disease in HFD mice infected with IAV. Our findings indicate that acetate has antiviral effects during IAV infection in both a human ex vivo lung model and pulmonary epithelial cells. Here, acetate prevents viral entry and affects the cellular metabolic state and antiviral response. Understanding these mechanisms could provide new targets for preventing and treating viral infections in individuals with diet-related health issues.

**Graphical Abstract:**

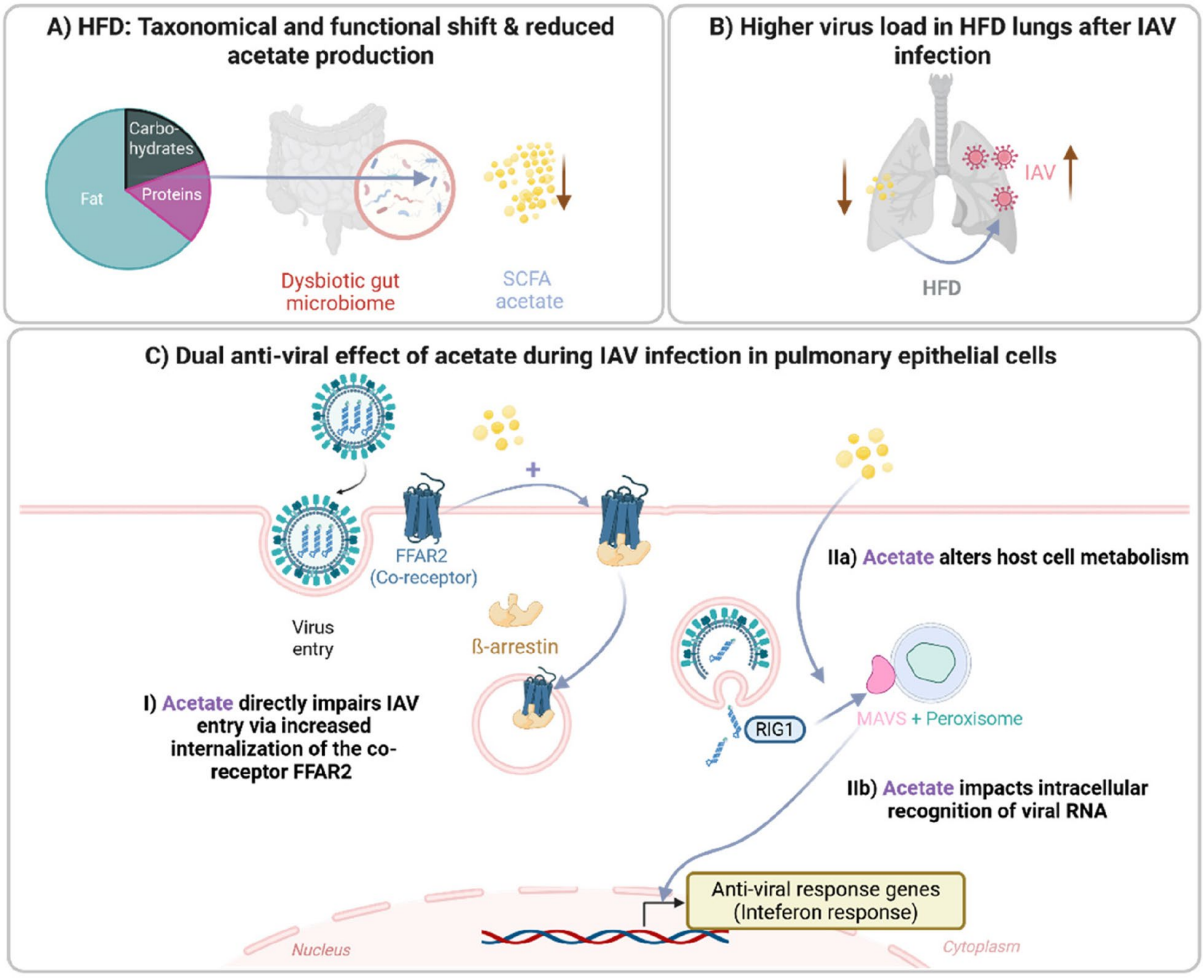

**Supplementary Information:**

The online version contains supplementary material available at 10.1186/s12964-025-02367-w.

## Background

The relationship between the human gut and its resident microbiome is linked to dietary habits, highlighting the impact of high-fat diets on this dynamic interplay [1]. The gut microbiome serves as an important source for various microbial metabolites, notably short-chain fatty acids (SCFAs) [2]. SCFAs are essential for modulating host energy homeostasis, and they are known to contribute to the development of metabolic disorders. Beyond their role as energy substrates for intestinal epithelial cells, SCFAs have emerged as key signaling molecules with widespread effects on various physiological processes and organs.

As byproducts of the microbial fermentation of dietary fibers within the gut, the consumption of diets rich in fat leads to the formation of potentially harmful microbial products [3]. Longitudinal studies have provided evidence that alterations in diet can significantly impact both the composition of the fecal microbiome and the profiles of microbial metabolites. In particular, high-fat diets (HFD), characterized by elevated levels of saturated and unsaturated fats, have been associated with strong modifications in gut microbial composition, often leading to dysbiosis. Interestingly, dysbiosis has been linked to changes in SCFA profiles, introducing a cascade of effects on various organs and physiological processes [4]. Taxonomical studies revealed an increased *Firmicutes* to *Bacteroidetes* ratio in a high fat uptake [5]. The former bacteria are more specialized in the calorie uptake and might be therefore increased. This explains why this ratio is also discussed as additional hallmark of obesity [6]. In summary, diet emerges as a critical determinant shaping the composition and functionality of the gut microbiota, thereby influencing the production of microbial metabolites.

The ability of SCFAs to cross the bloodstream as water-soluble molecules makes them important mediators in the complex interaction between the gut and other organs, including the lungs [7]. Considering this dynamic interplay, the gut-lung axis is of particular importance in the context of respiratory infections [8]. Also, influenza virus infections are reported to affect the intestinal microbiota, with a related injury to intestinal cells [9]. Furthermore, microbial metabolites are considered to exhibit antiviral efficacy through their interaction with the interferon response [9, 10].

Influenza virus infections contribute to significant morbidity and mortality worldwide. Beyond the multifaceted landscape of factors influencing virus susceptibility and severity, obesity is one of the discussed contributors [11]. Additionally, different dietary habits have been considered as factors impacting the complex interplay between viral pathogenesis and host physiology [12–14]. Among these factors, SCFAs play a crucial role, exerting their intracellular effects through three primary pathways: transporter-mediated entry via monocarboxylate transporters (MCTs) or aquaporins, endocytosis, and interaction with the SCFA receptors, g-protein coupled receptors (GPCRs), such as free fatty acid receptor 2 (FFAR2, also known as G protein-coupled receptor GPR43).

Our study aims to investigate the connections between modifications in the gut microbiome following a HFD, shifts in SCFA production, and their implications for the gut-lung axis during IAV infections. Utilizing an HFD mouse model, we observed significant modifications in gut microbiota composition, including an increase in the *Firmicutes/Bacteroides* ratio and a reduction in SCFA production-related genes, particularly acetate. We then infected HFD mice with IAV, which resulted in higher pulmonary virus titers and sustained microbiome alterations. To further understand the role of acetate, we conducted experiments using a human ex vivo lung model and pulmonary epithelial cells. We found that acetate exerted an antiviral effect, reducing IAV titers in both models. This effect was dependent on the FFAR2 receptor, known to be involved in viral entry, and intracellular signaling. Mechanistically, acetate reduced virus entry by promoting FFAR2 internalization and also influenced the early recognition of viral RNA via RIG-1, thereby enhancing the interferon response. In vitro,* the* antiviral effect of acetate also relied on the presence of interferon-γ-induced protein 10 (IP-10, *CXCL10*).

To the best of our knowledge, this is the first study to demonstrate a direct effect of acetate on influenza virus replication. Furthermore, we provide novel mechanistic insights into the role of FFAR2 in this context. Specifically, we show for the first time that acetate modulates the dynamics of the FFAR2 receptor, including its internalization. While previous studies have linked short-chain fatty acids to antiviral responses in RSV or RV models, our findings uncover a unique and previously unrecognized mechanism by which acetate influences host–pathogen interactions during influenza virus infection through FFAR2 signaling. Our findings suggest that the reduced acetate production in HFD-accompanied by an obese weight status may be a key factor in the increased severity of respiratory infections.

## Methods

### Virus strains and propagation

In vivo infections were conducted using the H1N1 influenza virus strain Influenza A/Jena/00084/16 (84/16) in normal-fat diet (NFD) and HFD mice, with ethics approval from the Jena University Hospital (no 2018 − 1263). This strain was isolated from a 37-year-old obese patient who died from IAV infection. After isolation, it was adapted through passaging and plaque purification in mice.

In vitro infections utilized the H1N1 influenza A virus/Puerto Rico/8/34 (PR8) strain in specific cell lines, including corresponding knock-out versions and human ex vivo lung slices. The virus was propagated in Madin-Darby canine kidney (MDCK) cells grown in Eagle minimum essential medium (EMEM) (ATCC, Manassas, VA, USA) supplemented with 10% fetal bovine serum (FBS) (PAN Biotech) and 1% Penicillin/Streptomycin (Sigma-Aldrich). Viral titers were assessed using a standard plaque assay to quantify plaque forming units (PFU) per ml [15].

### HFD mouse model and infection with IAV

The animal experiment was approved by the Office for Consumer Protection of Thuringia (TV-Nr: 02–018/16). Female BALB/cJRj mice (5 weeks old, JANVIER) were fed a HFD (ssniff Spezialdiäten GmbH) as previously described [16]. After an initial one-week adaptation phase, animals were randomly assigned to either the NFD or the HFD groups. The indicated timepoint before week 1 (0 weeks) represents the baseline time point after the one-week adaptation phase, with body weight normalized to 100%.

A total of 41 animals were allocated to two experimental groups: NFD (*n* = 21) and HFD (*n* = 20). Blood glucose levels were assessed via tail vein puncture by using ACCU Check (Roche) from 21 NFD and 20 HFD mice after 12 weeks of dietary period. After the dietary period, NFD (*n* = 21) and HFD (*n* = 20) mice were anesthetized with 2% isoflurane and intranasally infected with 10^6^ PFU of Influenza A virus (84/16) diluted in NaCl. After infection, NFD and HFD feeding was continued unchanged throughout infection and observation periods; diets were not switched back to NFD.

In the NFD group, 6 mice served as mock-infected controls, 3 mice were sacrificed on day 2 post-infection, and 9 mice were observed until day 21. Additionally, 3 animals had to be euthanized prematurely in accordance with animal welfare regulations due to exceeding the predefined humane endpoint criteria based on clinical scoring. In the HFD group, 6 mice were assigned as mock controls, 6 mice were sacrificed on day 2 post-infection, and 7 mice were monitored until day 21. One animal in this group was euthanized early due to severe clinical deterioration.

At day 2 post infection (dpi), the following groups were sacrificed for early response analysis and virus titration: 6 NFD mock-infected mice, 6 HFD mock-infected mice, 3 NFD Influenza A-infected mice and 6 HFD Influenza A-infected mice. The remaining animals of the NFD (*n* = 12) and HFD (*n* = 8) groups were monitored for up to three weeks post infection to assess longitudinal parameters. Fecal pellets were collected from all animals at 2 dpi and 21 dpi and from each group 3 samples were analyzed by shotgun transcriptomic.

### H&E staining and histoscoring

To analyze histopathological changes due to IAV infection in lung tissue, the left lung lobe was fixed in 3.5% formalin and processed through a series of ethanol and xylene for dehydration, then embedded in paraffin. Sections of 4 μm were cut with a microtome and stained with hematoxylin and eosin (H&E) to evaluate pulmonary pathology and cellular infiltration based on specific histoscore parameters (Supplemental Tab. S2) [17]. Sections from mock and mice at 2 and 21 days dpi were examined microscopically, and representative picture were captured with Axio Observer.Z1 (Zeiss) and analyzed with ZEN 2.6 (blue edition) (Zeiss).

### SCFA analysis of murine and human serum

Whole blood was extracted from mice post-dissection, and sera were obtained by centrifugation. SCFAs from NFD and HFD mice were quantified using a Mouse Short-Chain Fatty Acids ELISA kit (amsbio), following the manufacturer’s instructions.

Human serum samples were commercially obtained from the Blood Bank of the “Bayrische Rotes Kreuz gemeinnützige GmbH” (Munich, Germany) of female donors with non-obese (*n* = 4) and obese (*n* = 6) body mass index (BMI) (Supplemental Tabel S2). The quantification and analysis of SCFA was done via GC-MS by Creative Proteomics (New York, USA) [18].

### Human ex vivo precision-cut lung slices (PCLS)

PCLS were prepared from human lung tissue as described previously [19]. Briefly, this model is established by filling lung tissue with a mixture of medium and agarose. Sections of well-filled regions were embedded in agarose and cut into slices of 300 μm thickness. Slices were then cultured in well plates at 37 °C, 5% CO2 for further treatments. Slices were pre-incubated with 260 µM sodium acetate (NaOAc, Sigma Aldrich, St. Louis, MO, USA) in DMEM (Gibco), supplemented with 1% Penicillin/Streptomycin (Lonza Group) for 2 days. The concentration of 260 µM was used according to work from Antunes et al. [20]. Subsequently, slices were infected with 10^6^ PFU/ml of IAV/H1N1/PR8 for 2 h. Next, the virus solution was replaced by DMEM (Gibco), containing 1 mM MgCl_2_, 0.9 mM CaCl_2_, and 30 ng TPCK-treated trypsin (Thermo Fisher Scientific, Waltham, MA, USA) for 2 days of infection. Supernatants were collected, virus titer was determined via plaque assay, and frozen at −80 °C. Slices were fixed for Immunofluorescent staining with 4% PFA for 1 h and stored in PBS until further staining.

### Cell lines, acetate stimulation, and infection

In vitro experiments were carried out with A549 wildtype cells (*A549*, abcam, Cambridge, UK), and corresponding KO cells lines either with global KO of FFAR2 (*FFAR2-KO*, ubigene, China) or IP-10 (*IP-10-KO*, abcam). Wildtype cells were cultured in DMEM (Sigma-Aldrich), FFAR2-KO in Ham’s F-12 K (Gibco), and IP-10-KO in DMEM-F12 (Gibco) all supplemented with 10% FCS, 1% Pen/Strep. After seeding, cells were stimulated with 260 µM for 24 h according to Antunes et al., set up in the corresponding cultivation medium. Subsequently, infection was carried out with the IAV/H1N1/PR8 strain at a multiplicity of infection (MOI) of 1. Briefly, cells were washed and incubated with the virus-dilution prepared in PBS supplemented with 1 mM MgCl2, 0.9 mM CaCl2, 0.2% bovine serum albumin (BSA, Sigma-Aldrich). After 30 min at 37 °C, 5% CO2, cells were washed and further incubated for 8 and 24 h in medium supplemented with 30 ng TPCK-treated trypsin.

### Determination of the virus load by plaque assay

Plaque assays were conducted to assess virus load from supernatants of in vitro experiments, ex vivo PCLS, and in vivo lung homogenates. MDCK cells were seeded at 80–90% density in 12-well plates. Specimens were diluted in DPBS with 0.2% BSA, 1 mM MgCl2, 0.9 mM CaCl2, and 100 U/ml Pen/Strep, followed by a 30 min infection at 37 °C with 5% CO2. Plaque medium included Minimal Essential Medium (MEM, Gibco), 0.01% DEAE-Dextran (Sigma Aldrich), and 0.2% NaHCO3 mixed with 0.9% agar (Oxoid). After three days, plaques were visualized with neutral red staining (Sigma).

### Cytokine determination and LDH assay

For lung homogenate measurements from the in vivo mouse experiment, the LEGENDplex™ Mouse Anti-Virus Response Panel was used. In vitro experiments measured IP-10 (also known as CXCL-10) with the LEGENDplex™ Human Proinflammatory Chemokine Panel 1. All assays followed the manufacturer’s instructions, were measured with the Symphony A1 (BD Bioscience), and analyzed using the LEGENDplex™ Data Analysis Software Suite. LDH levels were measured using the CyQUANT LDH cytotoxicity assay kit (Thermo Fisher Scientific) according to the manufacturer’s instructions.

Immunofluorescent staining of ex vivo lung slices

The fixed tissue slices were stored in PBS until staining at 4 °C. Next, slices were permeabilized with 0.1% Triton-X (Carl Roth GmbH, Karlsruhe, Germany) for 1 h at room temperature (RT). After blocking in 3% BSA in PBS for 2 h at RT, slices were stained with mouse Influenza A Matrix Protein antibody-GA2B (1:100, Bio-Rad Laboratories, Hercules, CA, USA) and rabbit CD68 Polyclonal antibody (1:200, VWR) overnight at 4 °C. Subsequently, slices were washed three times with PBS and incubated with Alexa Fluor^®^ 488 AffiniPure Goat Anti-Mouse IgG (H + L) (1:500, Jackson ImmunoResearch), CyTM3 AffiniPure Donkey Anti-Rabbit IgG (H + L) (1:500, Jackson ImmunoResearch), together with AlexaFluor™Plus 647 Phalloidin (1:400, Thermo Fisher Scientific) for 2 h at RT. Slices were then mounted with DAPI Fluoromount-G (Southern Biotech) and imaged with the AxioObserver Z.1 + Apotome 2 (Carl Zeiss AG, Oberkochen, Germany).

### Lipid mediator measurement by UPLC-MS-MS

Lipid mediators were determined from supernatants in vitro and ex vivo. Briefly, 1 mL of ice-cold methanol with 10 µL deuterated LM standards (200 nM d8-5 S-HETE, d4-LTB4, d5-LXA4, d5-RvD2, d4-PGE2, and 10 µM d8-AA; Cayman Chemical/Biomol GmbH, Hamburg, Germany) was added to lung slice supernatants and A549 cells. After at least 60 min at −20 °C for protein precipitation, samples were centrifuged (1200 g, 4 °C, 10 min), and the supernatant was diluted with 4 mL acidified H2O (pH = 3.5). Sample preparation followed a published procedure [21]. For LM extraction, solid-phase cartridges (Sep-Pak^®^ Vac 6 cc 500 mg/6 mL C18; Waters, Milford, MA) were cleaned with 6 mL methanol and prepared with 2 mL H2O for loading. After sample addition, columns were washed with 6 mL H2O and 6 mL n-hexane, and LMs were eluted with 6 mL methyl formate. The eluates were dried in a solvent evaporation system (TurboVap LV, Biotage, Uppsala, Sweden) and reconstituted in 100 µL methanol-water (50/50). The LM profile was analyzed with an ACQUITY™ UPLC system (Waters, Milford, MA) and a QTRAP 5500 Mass Spectrometer (ABSciex, Darmstadt, Germany) with a Turbo V™ Source and electrospray ionization. LM separation used an ACQUITY UPLC^®^ BEH C18 column (1.7 μm, 2.1 mm × 100 mm; Waters, Eschborn, Germany) at a flow rate of 0.3 mL/min at 50 °C. The mobile phase comprised methanol-water-acetic acid (42:58:0.01) with methanol content increasing to 86:14:0.01 over 12.5 min and then to 98:2:0.01 for 3 min. The QTrap 5500 operated in negative ionization mode, using multiple reaction monitoring (MRM) with a 60-sec time frame linked to information-dependent acquisition. Optimized LM parameters (CE; Collision Energy, EP; Entrance Potential, DP; Declustering Potential, CXP; Collision Cell Exit Potential) were used with a curtain gas pressure of 35 psi. External standard means (Cayman Chemical/Biomol GmbH, Hamburg, Germany) confirmed LMs’ retention time and six diagnostic ions. Calibration curves for LM quantification showed r2 values of 0.998 or higher, with defined limits of detection for each LM.

### FFAR2 internalization and trafficking analysis

HEK293 cells were obtained from DSMZ Germany (ACC 305) and cultured at 37 °C with 5% CO2 in DMEM (Sigma-Aldrich, Germany), supplemented with 10% FCS and 1% penicillin and streptomycin. For live-cell confocal microscopy, HEK293 cells were transfected with β-arrestin2-YFP with or without FFAR2-mTurquoise using Effectene Transfection Reagent and seeded onto poly-D-lysine coated glass cover slips. Images were acquired at the Leica SP8 microscope in a 1024 × 1024 pixel format, using a 63x objective. mTurquoise was excited at 442 nm and YFP at 514 nm, with images taken before and after stimulation with 10 mM acetate from at least three independent experiments.

The BRET assay was conducted as described [22]. Cells were transfected with FFAR2-NanoLuc, β-arrestin2, CAAX-YFP, or FYVE-mNeonGreen; or FFAR2, β-arrestin2-NanoLuc, CAAX-YFP, or FYVE-mNeonGreen. Transfected cells were seeded in poly-D-lysine coated 96-well plates as triplicates. Cells were washed, and NanoLuc substrate furimazine (Promega, Germany) was added 1:3,500 in measuring buffer. Acetate-dependent BRET change was measured using a SynergyNeo2 plate reader. Baseline measurements lasted three minutes, followed by 30 more minutes after acetate stimulation. Final Δ net BRET change was derived from the corrected BRET change divided by the vehicle control. Results are presented as mean of at least three independent repetitions ± SEM.

### DNA extraction and Shotgun metagenomic sequencing of fecal pellets

Fecal pellets were collected before and after the diet, as well as on days 2 and 21 post infection (p.i.), then shock-frozen in liquid nitrogen and stored at −80 °C. DNA extraction was performed using the QIAmp PowerFecal Pro DNA Kit (Qiagen) according to the manufacturer’s guidelines. Library construction and shotgun metagenomic sequencing were conducted by Novogene UK. Genomic DNA was randomly fragmented, prepared with Illumina adapters, and sequenced on Illumina platforms.

Bioinformatic analysis included data quality control, metagenomic assembly, gene prediction via MetaGeneMark, and taxonomy annotation using the microNR database. Functional annotation was performed by comparing with KEGG and CAZy databases. Metastat’s multivariate statistical analysis was employed to explore differences in species and functional composition between groups.

### RNA extraction mRNA transcriptomic analysis of A549 cells

For transcriptomic analysis, RNA from A549 wildtype cells was extracted using the RNeasy Mini Kit (Qiagen) following stimulation and infection. Library construction, Illumina sequencing, and bioinformatics were performed by Novogene. After quality checks, data were aligned to the human reference genome with HISAT2, and gene expression was estimated in FPKM. Heatmaps of significantly differentially expressed genes were created using GraphPad Prism Version 9.

### Statistical analyses and scheme design

Data are presented as mean ± SD from at least three replicates with up to four significance levels. Boxplots show the median of at least three biological replicates with interquartile range. Statistical analyses were performed using GraphPad Prism v 9.0, as detailed in the figure legends. Graphical schemes were created with Biorender.com.

## Results

### HFD results in a distinct obese phenotype with a shift in gut microbiome composition

**Fig. 1:**
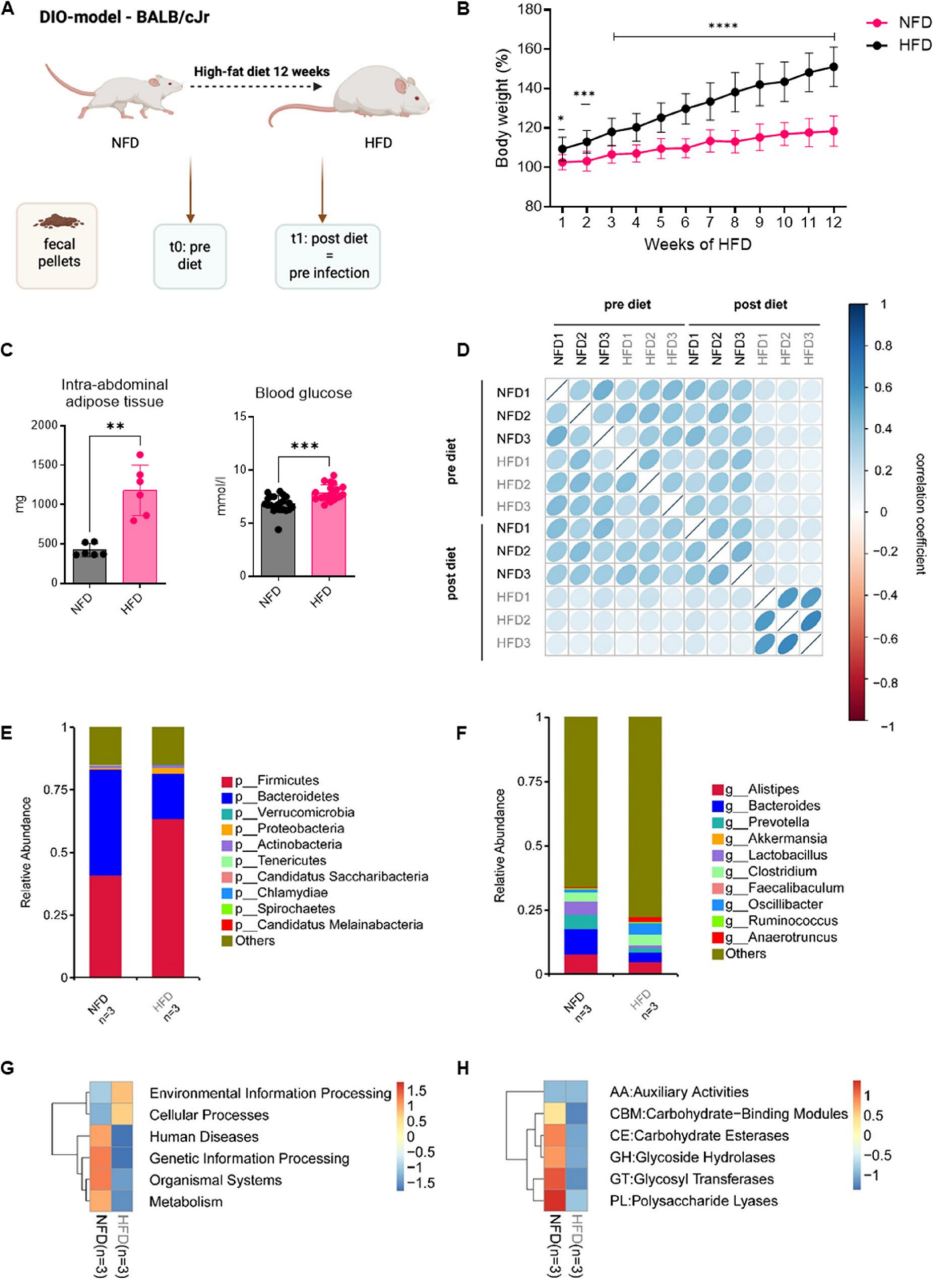
HFD results in a distinct obese phenotype with a shift in gut microbiome composition. Schematic representation of the in vivo diet-induced obesity (DIO) model with a 12-week HFD and collection of fecal pellets before and after the dietary period (**A**). The 12-week dietary period increased relative body weight (**B**) (NFD: *n* = 21, HFD: *n* = 20) with higher intra-abdominal adipose tissue mass (NFD: *n* = 6, HFD: *n* = 6) and blood glucose levels after the diet (NFD: *n* = 21, HFD: *n* = 20) (**C**). Values in panels B and C are displayed as mean ± SD. Gene correlation after shotgun metagenomics sequencing of fecal pellets shows correlation coefficients pre-diet and post-diet (**D**). HFD caused a taxonomical shift in gut microbiome composition at grouped phylum (**E**) and genus (**F**) levels, displayed as heatmaps with each group (*n* = 3). Additionally, it altered the functional profile after genetic annotation to the KEGG database level 1 (**G**) and CaZy level 1 (**H**), shown as absolute value of “Z” representing distance from raw value to population mean in SD units. *P < 0.05, **P < 0.01, ***P < 0.001, ****P < 0.0001. P calculated by 2-way ANOVA Šídák’s multiple comparisons test (**C**) and Mann-Whitney test (**D**)

The diet-induced obesity model (DIO) was carried out with female BALB/c mice, which were fed on an HFD for 12 weeks, as previously described (Fig. 1A) [16]. The diet consisted of approximately 21% metabolizable energy (ME) from carbohydrates, 19% from protein, and 60% from fat, signifying higher fat and lower carbohydrate content compared to the NFD (62% carbohydrates, 20% protein, 5% fat) (Supplemental Tab. S1, Supplemental Fig. S1A). Throughout the dietary period, body weight was monitored weekly, and fecal pellets were collected before and after the diet (Fig. 1A). The relative body weight showed a significant increase in the cohort of mice on the HFD (Fig. 1B). Moreover, mice on the HFD exhibited a notable increase in intra-abdominal adipose tissue mass and blood glucose levels (Fig. 1C).

To study the effects of the HFD on the microbial environment of the gut, we conducted shotgun metagenomic sequencing using fecal samples, resulting in information about the taxonomic variety of microbes and their functions. We analyzed fecal samples before starting the diet (pre-diet) and after 12 weeks of either NFD or HFD (Fig. 1D) and found that all mice receiving the same diet exhibited a positive correlation in gene expression. Notably, mice that transitioned from NFD to HFD displayed an even stronger positive correlation, with a mean correlation coefficient of 0.585, compared to NFD mice, which had a mean correlation coefficient of 0.365 (Fig. 1D).

The analysis of genes in the mice cohort showed that those receiving the HFD had more bacteria from the phyla *Firmicutes* and *Proteobacteria* and fewer from *Bacteroidetes* (Fig. 1E). Additionally, the numbers of bacteria from the *Bacteroides*,* Prevotella*, and *Lactobacillus* genera decreased, while *Anaerotruncus* increased (Fig. 1F). Further analysis of the murine fecal pellets revealed differences in gene abundance between the HFD and NFD cohorts. Genes related to general metabolism, organismal systems, genetic information processing, and human diseases were less abundant in the HFD mice, while genes linked to environmental information processing and cellular processes were more abundant (Fig. 1G). Comparing the abundant genes with the CAZy database showed that the HFD group had reduced expression of genes related to carbohydrate-active enzymes compared to the NFD group (Fig. 1H).

Hence, feeding mice with HFD not only resulted in an increase in body weight, abdominal adipose tissue mass, and blood glucose levels. Moreover, this specific diet led to taxonomical and functional shifts in the microbial community within the gut.

### Elevated virus titers and increased pulmonary inflammation in HFD mice infected with IAV

**Fig. 2:**
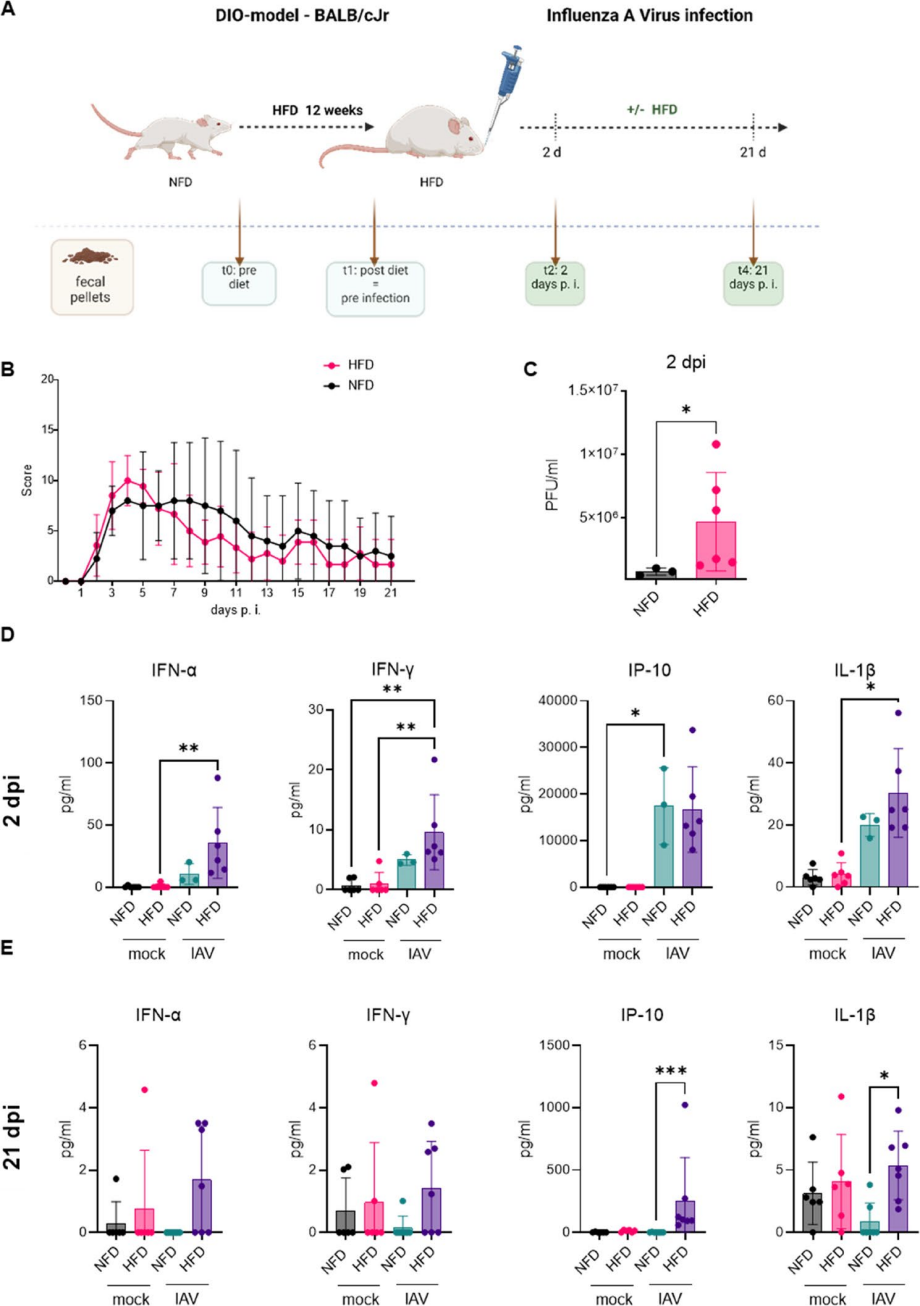
Elevated virus titers and increased pulmonary inflammation in HFD mice infected with IAV. Schematic overview of intranasal infection of NFD (*n* = 15) and HFD (*n* = 14) mice with IAV, including organ harvest and fecal pellet collection after 2 and 21 days (**A**). Scoring values for NFD and HFD mice across 3 weeks p.i. (**B**). HFD mice (*n* = 6) exhibited higher active virus particles in lung tissue at day 2 p.i. compared to NFD mice (*n* = 3) (**C**). HFD mice showed altered pulmonary cytokine profiles at acute phase (2 days post infection (dpi), HFD *n* = 6, NFD *n* = 3) (**D**) and long-term (21 dpi, HFD *n* = 6, NFD *n* = 6) (**E**) compared to mock NFD (*n* = 6) and HFD (*n* = 6) mice. Values are mean ± SD. *P < 0.05, **P < 0.01, ***P < 0.001, calculated by Kruskal-Wallis test, Dunn’s multiple comparison test (**C**-**E**)

After the dietary period, mice were infected with an H1N1 IAV strain, obtained from an obese patient at the Jena University Hospital. Before infecting NFD and HFD mice, the virus strain was modified through passaging, plaque purification, and isolation, as previously described [16]. Mice from both dietary groups (w or w/o IAV) were euthanized 2 dpi to assess the acute phase of infection, and 21 dpi to evaluate long-term changes in mice after (Fig. 2A). Throughout the infection period, all mice were monitored daily, and body weight and burden score values were recorded. The scoring system not only considers body weight but also includes additional parameters to assess the severity of the disease in mice infected with IAV [16]. The burden score values for NFD mice decreased from day 5, reaching an average maximum at day 4 p.i. Interestingly, HFD mice reached maximal score values at day 4 p.i. and maintained them at the same level until day 10 p.i. (Fig. 2B). H&E staining was performed on the left lung lobe of mice both before infection and at 2 and 21 dpi (Supplemental Fig. S1B). In addition to the qualitative observations of pulmonary inflammation within the tissue, a histoscoring system was utilized to quantify these findings (Supplemental Tab. S2). At both time points, the infection led to an increased histoscore; however, no clear trend was observed between mice on NFD and on a HFD (Supplemental Fig. S1C). At 2 dpi, the number of infectious virus particles detected in homogenized lung tissues was significantly higher in mice on a HFD (Fig. 2C). Additionally, the levels of pro-inflammatory mediators in the lung homogenates of infected mice at 2 dpi were elevated in HFD mice, particularly interferon (IFN)-α, IFN-γ, and interleukin (IL)−1β (Fig. 2D).

By day 21 p.i., no infectious virus particles were found in the lungs of either the non-obese or obese mice. However, lung tissue from HFD mice produced significantly higher amounts of IP-10 (encoded by *CXCL10*) and IL-1β. The levels of the interferons, IFN-α and IFN-γ, remained elevated in the lungs of HFD mice three weeks after infection (see Fig. 2E). Moreover, the levels of IL-10, C-X-C Motif Chemokine Ligand 1 (CXCL1), Regulated and Normal T cell Expressed and Secreted (RANTES), and Granulocyte-Macrophage Colony-Stimulating Factor (GM-CSF) were predominantly increased in the infected HFD group (Supplemental Fig. 2). Interestingly, a similar trend toward increased pulmonary inflammation was also noted in uninfected HFD mice, although it did not reach statistical significance (Fig. 2E).

In summary, infection of HFD mice led to significantly increased virus loads in the pulmonary system accompanied by increased inflammation at an acute state of infection and after three weeks.

### Dysregulated gut microbiome taxonomy and SCFA production in infected HFD mice

**Fig. 3:**
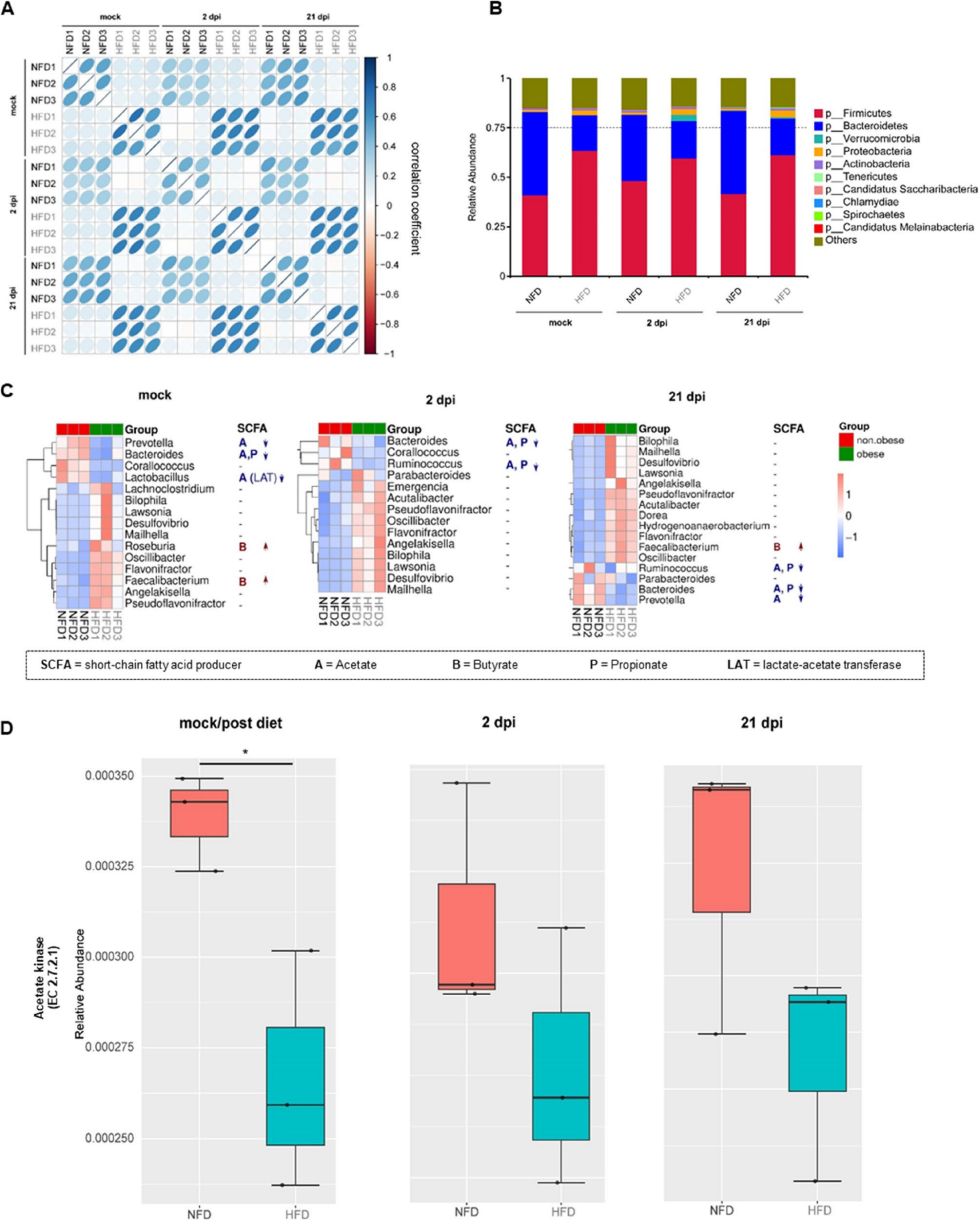
Dysregulated gut microbiome taxonomy and SCFA production in infected HFD mice. Gene correlation analysis after shotgun metagenomics from fecal pellets at 2 and 21 days post infection (dpi) from NFD and HFD depends on diet (**A**). Changes in microbiota composition during infection depend on diet at the phylum level (**B**). Heatmaps from LefSe analysis show significantly different genera related to SCFA production between HFD (*n* = 3) and NFD (*n* = 3) mice at mock, 2 dpi, and 21 dpi. The heatmap values are standardized Z values for relative abundance (**C**). Reduced expression of Acetate kinase (EC 2.7.2.1) was significant in mock mice after 12 weeks of diet, shown as relative abundance (NFD, *n* = 3; HFD, *n* = 3). *P < 0.05 & *Q < 0.05 calculated via Metastats (**D**)

The correlation analysis of bacterial genes isolated from the fecal pellets of both dietary cohorts of uninfected and infected mice at day 2 and 21 p.i. showed a similar pattern to the one observed after an HFD (Fig. 3A). Furthermore, the increased ratio of *Firmicutes* to *Bacteroidetes*, previously observed in mice after the HFD, was still present during infection. Additionally, the phylum *Proteobacteria* was increased in HFD mice before, during acute infection, and after 21 days. Bacteria of the phylum *Verrucomicrobia* were predominantly increased in infected obese mice after 2 days (Fig. 3B).

We studied the impact of diet and IAV infection on specific metabolite-producing bacteria. For this, we focused on bacterial genera known to produce SCFA such as acetate, propionate, and butyrate before and during infection, as described by Koh et al. [23]. LefSe (linear discriminant analysis (LDA) Effect Size) was used to identify genomic features (genes, pathways, or taxa) that characterize the differences between various conditions (Fig. 3C). In the absence of infection, HFD mice displayed reduced levels of *Prevotella*,* Bacteroides*, and *Lactobacillus* bacteria, which are known to produce acetate and propionate. However, butyrate-producing genera *Roseburia* and *Faecalibacterium* increased. *Bacteroides* and *Ruminococcus*, which also produce acetate and propionate, decreased in obese mice at day 2 and 21 p.i. Furthermore, *Prevotella*, which can also produce acetate, decreased. *Faecalibacterium* bacteria, known for producing butyrate, increased at day 21 p.i. (Fig. 3C).

During the infection, genes related to lipid metabolism remained reduced in mice on a HFD compared to mice fed with a NFD. Interestingly, genes associated with human diseases increased in HFD mice during the acute phase of infection, at 2 dpi (Supplemental Fig. S3A). Analysis of CAZy showed a similar reduction as seen after the diet, with a decreased expression of genes in all six categories (Auxiliary Activities (AA), Carbohydrate Binding Molecules (CBM), Carbohydrate Esterases (CE), Glycoside Hydrolases (GH), Glycosyl Transferases (GT), Polysaccharide Lyase (PL)) in HFD-receiving mice, at 2 dpi and 21 dpi, except for AA, which was comparatively increased at day 2 p.i. (Supplemental Fig. S3B).

Despite the limited volume of serum available in mice for SCFA detection, we successfully quantified acetate levels in a reduced number of uninfected normal-fat diet (NFD) and high-fat diet (HFD) mice after the dietary period. Although the levels were decreased, this change did not reach statistical significance (Supplemental Fig. S3C). To evaluate genes related to the production of SCFAs, we conducted a KEGG analysis focused on SCFA-producing pathways, represented as relative abundances (Supplemental Fig. S3D, Fig. 3D). Notably, the phosphate acetyltransferase-acetate kinase pathway, which is responsible for acetate synthesis, showed a significant reduction in uninfected mice on HFD (Supplemental Fig. S3D). This effect was also observed during infection, although it did not reach statistically significant levels. Additionally, at the enzymatic level, acetate kinase—a key enzyme involved in acetate synthesis—exhibited similar trends (Fig. 3D).

Therefore, after HFD and during infection, the gut microbiome of HFD mice showed alterations at a taxonomical and functional level. Specifically, the production of SCFAs differed, with the relative abundance of genes associated with acetate synthesis being lower in mice on the HFD.

### Reduction in virus titer in infected human ex vivo lung slices pre-treated with acetate

**Fig. 4:**
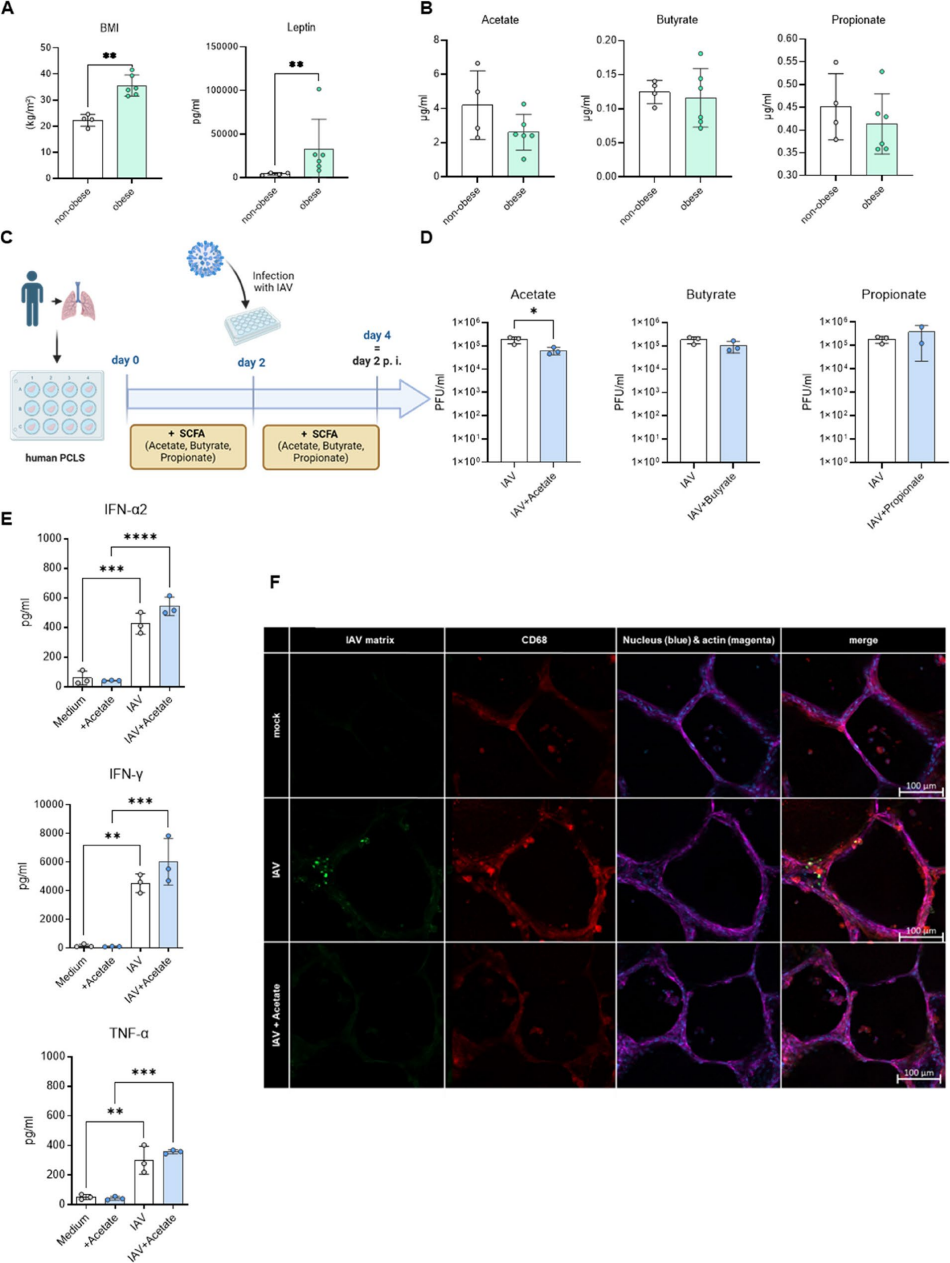
Reduction in virus titer in infected human ex vivo lung slices pre-treated with acetate. Healthy female donor cohorts (according to Supplemental Tab. S3) were characterized by a normal BMI (< 25 (kg/m²), *n* = 4) and an increased BMI for obese individuals (> 30, *n* = 6) and elevated systemic leptin levels (**A**). There was a trend towards reduced systemic acetate (*p* = 0.2189, 𝑅2 = 0.3376), butyrate (*p* = 0.6727, 𝑅2 = 0.02703), and propionate (*p* = 0.4392, 𝑅2 = 0.1009) levels (**B**). Schematic overview of human ex vivo lung slices (PCLS) treated with SCFAs for 2 days and subsequent infection with IAV for 2 days (**C**). Acetate treatment of lung slices resulted in lower active virus particles, or plaque forming units (PFU/ml) in supernatants (**D**). Solely IAV infection increased levels of IFN-α2, IFN-γ, and TNF-α, while acetate pretreatment slightly enhanced cytokine production (**E**). Reduced IAV matrix protein (green) expression in infected lung slice pre-treated with acetate within the alveolus, co-stained with CD68 (red), DAPI (blue), and Phalloidin (purple) (**F**). Scale bar = 100 μm. Values in panels A, B, D, and E are mean ± SD. *P < 0.05, **P < 0.01, ***P < 0.001, ****P < 0.0001, P calculated by Mann-Whitney test (**A**, **D**), unpaired t-test with Welch’s correction (**B**), one-way ANOVA, Tukey’s test (**E**)

In order to analyze the relationship between diet and gut metabolites in relation to infection in humans, we studied the blood of healthy female donors, categorizing them as non-obese or obese based on their BMI [24]. Donors with a BMI of < 25 were classified as “non-obese,” while individuals with a BMI of > 30 were classified as obese [25] (Supplement Tab. S3). Elevated BMI was associated with higher higher serum leptin levels and increased age (Fig. 4A, Supplemental Fig. S4A). Donors were carefully selected within the same age range (30–45 years) to limit age-related variability. Besides leptin, also other cytokines and adipocytokines were determined in the serum, but with no significant trends (Supplemental Fig. S4B). Additionally, we measured levels of the SCFAs acetate, butyrate, and propionate. Acetate exhibited the most notable decrease in the blood of obese donors compared to non-obese donors (Fig. 4B).

To study the potential positive effect of SCFAs on the human lung, we used an already established human ex vivo PCLS model [19]. The lung slices were pre-incubated with specific concentrations of individual SCFAs (acetate, butyrate, and propionate) for 2 days and then infected with an H1N1 strain (PR8/34) for additional 2 days. After the infection, SCFAs were reintroduced into the medium.(Fig. 4C). Interestingly, only the lung slices stimulated with acetate showed a significant reduction in the number of infectious virus particles. The treatment with butyrate and propionate did not show any significant alterations (Fig. 4D). These findings were consistent with an early significant increase in the cytokines IFN-α2, IFN-γ, and tumor necrosis factor (TNF) - α due to infection. Slices additionally treated with acetate did lead to a slight increase of IFN-α and IFN-γ, yet reaching no significant level (Fig. 4E). To confirm these findings, we performed immunofluorescence staining, which also showed a qualitative reduction in virus particles in the alveoli after treatment with acetate in combination with infection (Fig. 4F).

Thus, obese, human, female donors also displayed dysregulated levels of SCFAs in their circulation, with the most noticeable impact seen in reduced acetate levels. This particular SCFA, when analysed during treatment in a human ex vivo system in combination with infection, demonstrated the most significant beneficial antiviral effect.

### Antiviral effect of acetate depends on FFAR2 activation and internalization dynamics in pulmonary epithelial cells

**Fig. 5:**
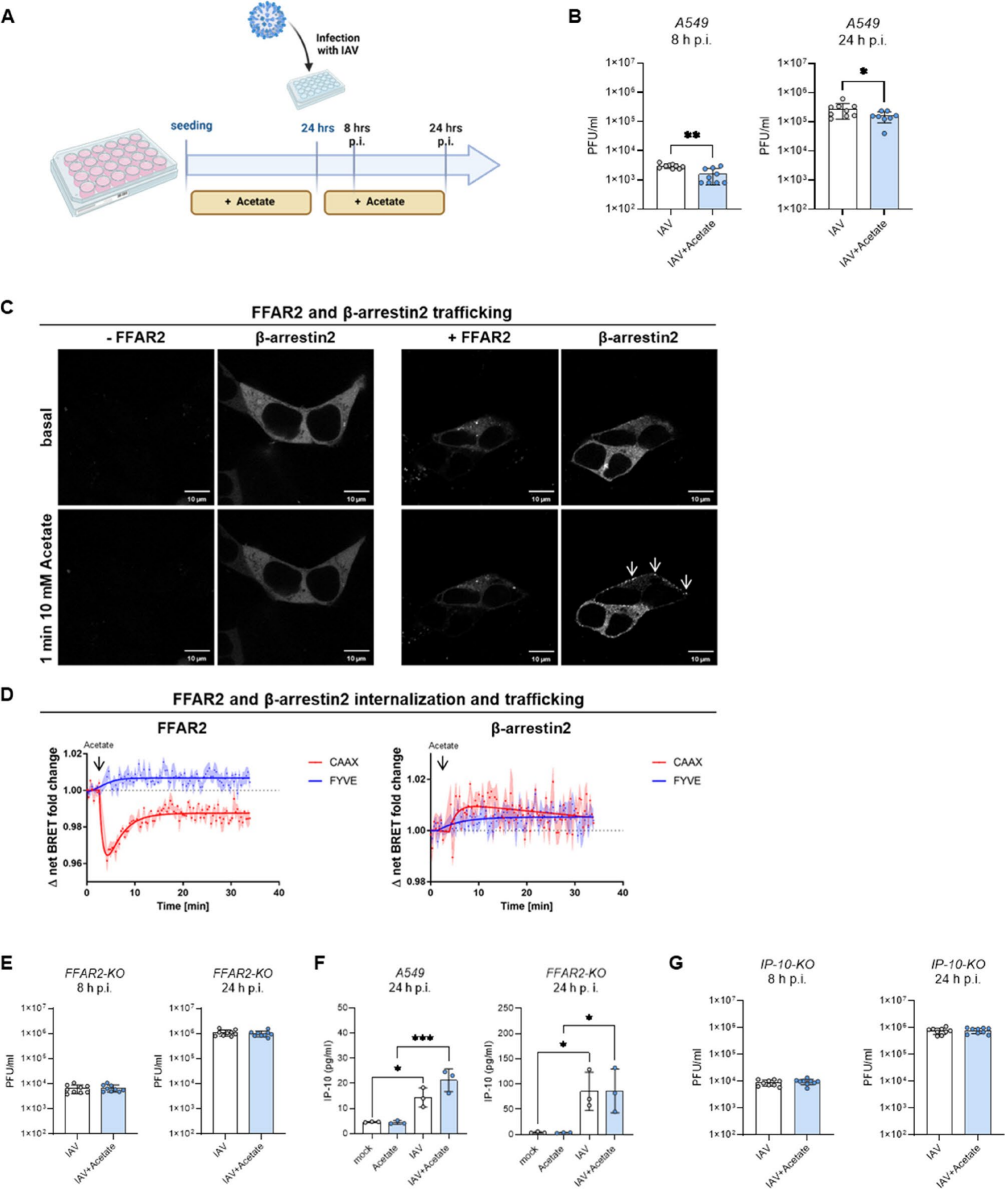
Antiviral effect of acetate depends on FFAR2 activation and internalization dynamics in pulmonary epithelial cells. Schematic representation of A549 cells pre-treated with acetate and infected with IAV for 8 and 24 h (**A**). Data represent *n* = 9 independent biological replicates per condition. Acetate treatment reduced active virus particles in the supernatant of infected wildtype A549 at 8 and 24 h post infection (hpi) (**B**). Acetate treatment increased ß-arrestin2 recruitment to the cell membrane (arrows) in HEK293 cells expressing FFAR2 compared to untreated and FFAR2-deficient cells (**C**). Furthermore, acetate stimulation caused FFAR2 translocation from the plasma membrane (CAAX) to early endosomes (FYVE) (left), while ß-arrestin2 also translocated to early endosomes (right) (**D**). Reduction of active virus particles was absent in A549 cellswith global FFAR2 receptor knock-out (**E**). IP-10 levels were increased in response to infection in supernatants of both A549 WT and FFAR2-KO cells, with a noticeable increase in WT cells at 24 hpi with IAV (*n* = 3) (**F**). Acetate pre-treatment on A549 with global knock-out of IP-10 did not affect the number of active virus particles after 8 or 24 hpi (*n* = 9) (**G**)

To elucidate the molecular mechanisms underlying the detected antiviral effect of acetate observed during IAV infection in the human lung ex vivo slices, we conducted similar stimulation and infection experiments in pulmonary epithelial cells (A549) for 8 h and 24 h of infection (Fig. 5A). The A549 cell line is known to mimic properties of the alveolar type II cell line, including the presence of lamellar bodies [26]. Stimulation with medium containing 260 µM acetate did not increase cytotoxicity at either 8–24 h (Supplemental Fig. S5A). Consistent with the results from ex vivo lung slices, acetate stimulation reduced virus titer determined in supernatants of A549 cells at both 8 and 24 h post infection (hpi) (Fig. 5B).

To further analyze the effect of acetate on the FFAR2 receptor, we examined the impact on receptor internalization in HEK293 cells, focusing on the ability to recruit β-arrestin2, an important mediator of GPCR endocytosis [27]. Live-cell confocal microscopy imaging revealed that β-arrestin2 is recruited to the cell membrane in an acetate-dependent manner. This recruitment depends on the presence of the FFAR2 since cells not expressing the receptor do not show any acetate-induced recruitment of β-arrestin2 to the plasma membrane (Fig. 5C).

To comprehensively investigate the internalization and early trafficking characteristics of the FFAR2 we conducted BRET assays utilizing the energy transfer between NanoLuc-tagged FFAR2 or β-arrestin2 and membrane-localized CAAX-YFP (prenylation sequence of KRas, plasma membrane) and FYVE-mNeonGreen (phosphatidylinositol 3-phosphate (PI3P) binding motive of endofin, early endosomes) constructs, as previously described [28]. The analysis revealed an acetate-dependent, rapid translocation of the FFAR2 from the plasma membrane (CAAX) to the early endosomes (FYVE). About ten minutes after acetate treatment, the relocation of FFAR2 to the plasma membrane reaches a plateau that does not return to the basal level, indicating reduced FFAR2 levels at the cell surface in the presence of acetate. Additionally, a part of the internalized FFAR2 stays at the early endosomes 30 min after acetate stimulation. In order for the FFAR2 to be internalized, adaptor protein β-arrestin2 is recruited to the plasma membrane (CAAX) and translocates to the early endosomes (FYVE), most probably together with the FFAR2 (Fig. 5D).

To analyze the effect of acetate and FFAR2 in the pulmonary epithelial cells during IAV infection, we repeated the stimulation and infection setup using A549 cells with a global knock-out of FFAR2 (*FFAR2-KO*). Interestingly, no reduction of virus titer was observed in acetate-treated *FFAR2-KO* cells, indicating that the antiviral effect of acetate during IAV infection is dependent on the GPCR FFAR2 (Fig. 5E).

Moreover, we aimed to investigate the possible influence on the interferon system, a crucial player in the antiviral response, by measuring the amount of IP-10 (encoded by *CXCL10*). This chemokine is known to be produced in response to influenza virus infection and plays a role in modulating the antiviral response [29]. In supernatants of both in vitro cell lines A549 WT and FFAR2-KO, infection with IAV resulted in a significant upregulation of IP-10 after 24 h of infection. Acetate treatment led to slightly increased levels of IP-10, although this increase was not statistically significant (Fig. 5F). In *FFAR2-KO* cells, this treatment did not show this trend. To assess the impact of the production of IP-10 on the reduced viral titer detected in A549 WT cells, absent in FFAR2 deficient cells, we repeated the same experimental set-up with infection in combination with acetate treatment on IP-10 deficient cells (*IP-10-KO*). Similar to *FFAR2-KO* cells, also *IP-10-KO* did not exhibit any reduction of active virus particles following acetate treatment, neither after 8 nor 24 hpi (Fig. 5G).

Taken together, acetate stimulation led to a significant reduction in viral titers at both 8 and 24 h post-infection in pulmonary epithelial cells. FFAR2 trafficking studies demonstrated that acetate promoted β-arrestin2–mediated internalization of the receptor. This antiviral effect was absent in A549 cells lacking either FFAR2 or the key interferon response mediator IP-10, suggesting both are essential for acetates mechanism during infection.

### Acetate treatment affects cellular metabolism during IAV infection

Based on the in vitro results with A549 WT and FFAR2-KO cells, we concluded the beneficial effect of acetate on IAV infection is dependent on the presence of the FFAR2 receptor. Next, we aimed to further elucidate the intracellular connection between acetate and IAV infection. Since the cytokine profile from infected and stimulated human ex vivo lung slices showed no distinct alterations with acetate treatment, we proceeded to determine pro-inflammatory lipid mediators derived from arachidonic acid. Previous work has shown that cyclooxygenase products, such as prostaglandin (PG) E_2_, PGF_2α,_ and thromboxane (TX) B_2,_ are produced by macrophages during infection with IAV [30]. In the complex human ex vivo lung slice model, IAV infection resulted in an upregulation of all three mediators, most prominently for PGE_2_ and PGF_2α_. However, the acetate pre-treatment did not affect the levels of these secreted markers. Interestingly, we could not detect any lipid mediators in the supernatant of infected A549 cells (Supplemental Fig. S5B).

Furthermore, we performed mRNA transcriptomic analysis to examine the alterations caused by acetate per se in A549 cells, focusing on the transcriptomic profile of pulmonary epithelial cells with and without infection. In vitro incubation for 24 h resulted in significant changes in the overall distribution of differentially expressed genes, with a notable upregulation of 126 and downregulation of 44 genes compared to the medium control (Supplemental Fig. S5C). KEGG annotation revealed a specific upregulation of various metabolic pathways, including those involved in steroid and terpenoid biosynthesis, which are known to have antiviral functions, as determined by mRNA-sequencing (Supplemental Fig. S5D) [31].

Interestingly, at 8 h p.i., genes connected to metabolic processes such as cholesterol metabolism and glycolysis/gluconeogenesis were significantly upregulated in cells pre-treated with acetate compared to the sole infection (Supplemental Fig. S5E). Strikingly, genes involved in peroxisome function were downregulated in the same condition (Supplemental Fig. S5F). The peroxisome is known to be involved in various processes of lipid metabolism and the generation of reactive oxygen species (ROS), in addition to its involvement in viral infections [32].

In summary, acetate did not affect the secretion of lipid mediators, such as PGE2. However, 24 h pre-incubation in the pulmonary epithelial cell model resulted in an altered cell metabolism, which was significantly visible at an early point of infection. Interestingly, genes related to the peroxisome, an organelle connecting cellular metabolism and viral processes, were reduced in epithelial cells treated with acetate and infected with IAV.

### Early antiviral effect of acetate during IAV infection in epithelial cells

In the next step, we analyzed transcriptomic data from infected A549 WT cells, focusing specifically on genes related to IAV response. At 8 hpi, the genetic profile of cells infected and pre-treated with acetate (IAVAc) showed alterations compared to unstimulated infection (IAV), adjusted to the responsible mock cells (Mock and Ac). Notably, genes including IP-10 (*CXCL10*), Interferon induced with helicase C domain (*IFIH*) 1, interferon regulatory factor (*IRF*) 1, *IRF3* or oligoadenylatsynthetase (*OAS*) 2 were not significantly upregulated when treated with acetate.

**Fig. 6:**
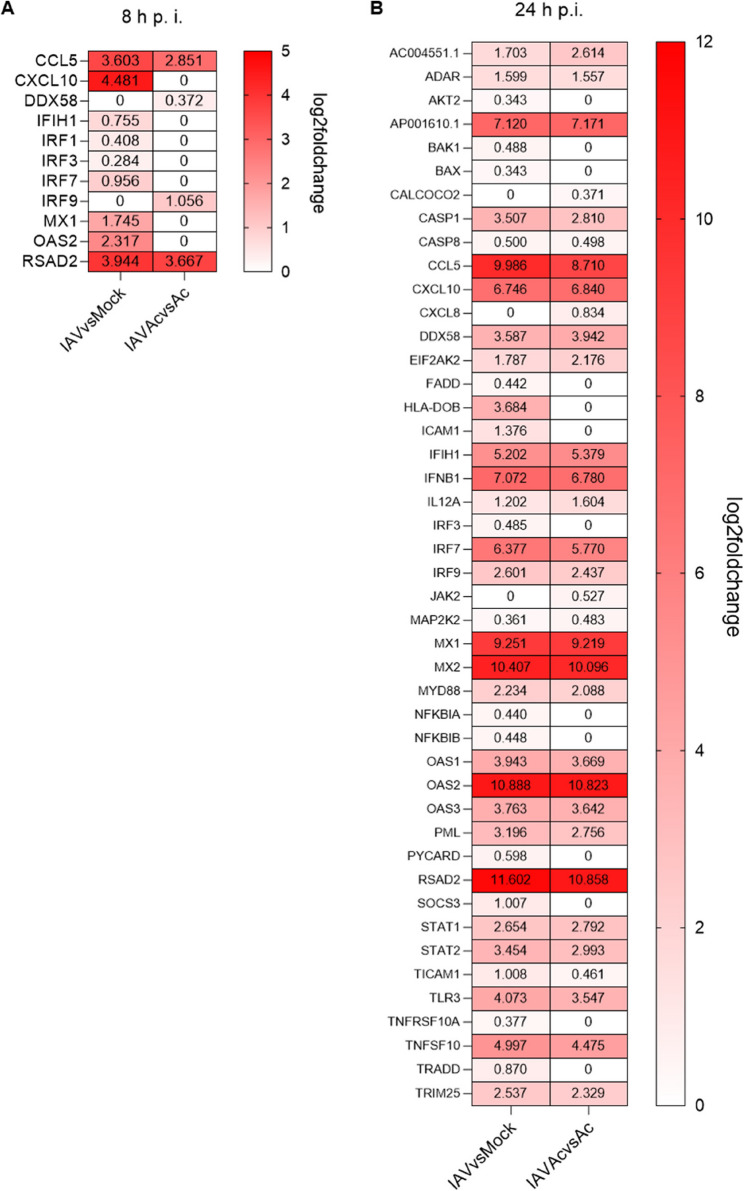
Early antiviral effect of acetate during IAV infection in epithelial cells in vitro. Heatmaps of differentially expressed genes at 8 h post infection (hpi) showed more upregulated IAV response genes in acetate pre-stimulated A549 WT cells compared to controls, except for *DDX58* (RIG-1) and I*RF9* (**A**). At 24 hpi, there was again an increase in upregulated genes in infected cells pre-treated with acetate compared to controls, except for *CALCOCO2*,* CXCL8*, and *JAK2* (**B**). Importantly, *CXCL10* is the gene encoding for IP-10

Further studying anti-virus-related proteins revealed that specific genes, including *DDX58* and *IRF9*, were upregulated after 8 h. *IRF9* and *DDX58* are central components of the type I interferon response and play key roles in early viral sensing and antiviral signaling. Our data indicate that their expression is enhanced in acetate-treated conditions during IAV infection, suggesting that acetate may potentiate early innate immune recognition of the virus. At the later time point of 24 h, there is a general downregulation of genes related to IAV infection (Fig. 6B).

When directly comparing IAVAc cells with IAV cells, various genes were significantly up- or downregulated at the early 8 h time point (Supplemental Fig. S6A). Specifically, after 8 h genes connected to toll-like receptor (TLR) and TNF signaling were significantly upregulated in IAVAc cells (Supplemental Fig. S6B). After 24 h of infection, the volcano plot—a type of scatter plot commonly used in statistics and bioinformatics to illustrate the results of differential expression or large-scale data comparisons—showed a significant upregulation and downregulation of various genes. This is represented in Supplemental Fig. S6C, which highlights the differentially expressed genes (DEGs).At this time point, the already observed reduced transcription of general IAV related was confirmed, with significantly regulated genes of the interferon and TNF signaling (Supplemental Fig. S6D).

## Discussion

Since the influenza virus pandemic of 2009, obesity has emerged as an independent risk factor for severe infection courses [33]. While the role of obesity in infectious disease outcomes is increasingly recognized, the precise mechanisms underlying this association remain poorly understood.

In line with previous murine studies, HFD feeding induced significant weight gain, increased adipose tissue mass, and elevated glucose levels [16]. Microbiome analysis revealed classical features of diet-induced dysbiosis, notably an increased *Firmicutes/Bacteroidetes* ratio and elevated levels of *Proteobacteria* [5, 6, 34]. These taxonomic shifts are hallmark indicators of an obese gut microbiome and are known to coincide with altered microbial functionality. Indeed, metagenomic analysis in our study showed reduced expression of microbial genes related to carbohydrate metabolism and carbohydrate-active enzymes (CAZymes). Given the drastically lower proportion of metabolizable energy from carbohydrates in HFD (21% vs. 60% in NFD), this downregulation likely reflects reduced fermentative activity in the gut, which in turn limits SCFA production [35].

Diet is a key factor shaping gut microbiota composition; however, genetic predisposition, host metabolic status, and environmental influences also contribute significantly to inter-individual variation in microbiota profiles [36]. This complexity must be taken into account when interpreting our findings, particularly in the context of obesity and metabolic syndrome, where multiple interacting factors may influence both microbiota composition and host immune responses.

Infection of obese (DIO) mice with IAV resulted in higher viral loads and enhanced pulmonary inflammation at early and late stages of infection, in line with earlier studies [16, 37–39]. These findings indicate that HFD not only compromises metabolic health but also increases susceptibility to severe respiratory viral disease. Of particular interest is our observation that the gut microbiome remained dysbiotic during IAV infection, with sustained increases in *Firmicutes* and *Proteobacteria*, as well as transient enrichment of *Verrucomicrobia* at 2 dpi. This indicates that infection does not restore microbial homeostasis but rather exacerbates the dysbiotic profile. We further observed a reduction in genes associated with acetate production across all infection stages in HFD-fed mice. Acetate, along with propionate and butyrate, constitutes the major SCFAs produced by anaerobic fermentation of dietary fibers by gut bacteria such as Bacteroides, Lactobacilli, and Eubacteria [40]. These SCFAs are absorbed into the circulation and have systemic effects, including modulation of immune responses in distant organs such as the lungs [41]. The decreased abundance of acetate-producing genes, consistent with reduced SCFA levels reported in HFD models, suggests an impaired gut-lung axis signaling in the context of obesity [42–44].

To assess whether the observed reduction in acetate-related gene pathways in the gut microbiome of HFD-fed mice was accompanied by altered metabolite levels, we directly measured acetate concentrations in serum samples from both diet groups. These measurements revealed a trend toward lower acetate levels in HFD-fed mice compared to their NFD-fed counterparts. Although the difference did not reach statistical significance, this observation aligns with prior reports demonstrating decreased microbial SCFA production in the context of high-fat feeding and reduced dietary fiber intake [42–44].

In an effort to increase the translational relevance of our findings, we extended our analysis to human samples. Specifically, we measured serum acetate concentrations in a cohort of healthy female donors stratified by body mass index (BMI) into normal weight and obese groups. Consistent with our murine data, we observed a trend toward lower systemic acetate levels in obese individuals. However, similar to the murine fecal measurements, these differences did not achieve statistical significance.

A notable limitation of our study is that neither the murine nor the human acetate measurements yielded statistically significant group differences. This is likely attributable to the high degree of inter-individual variability in SCFA levels, which has been extensively documented in the literature [45]. A limitation of our study is that the human data are restricted to SCFA levels and do not provide direct causal evidence for the observed associations. Several potential confounders must be taken into consideration: (i) dietary habits, which critically influence SCFA production, were not systematically recorded. (ii) Likewise, gut microbiota composition is highly individual and shaped by factors such as age, environmental exposures, and antibiotic use. Due to this inherent variability, and in line with previous studies, we deliberately refrained from pursuing large-scale cohort analyses solely aimed at achieving statistical significance. Instead, we focused on the investigation of acetate on IAV infections.

We further acknowledge that several aspects of our study, particularly experiments involving human donors and microbiome sequencing with a limited number of animals are underpowered. While the trends observed are consistent and biologically plausible, the small sample sizes limit the statistical confidence and generalizability of our findings.

To evaluate the impact of acetate on human lung tissue during infection, we took advantage of the previously established human ex vivo lung model [19]. Again, acetate pretreatment for 2 days resulted in a reduced antiviral effect at 2 dpi when transferring the results from the murine HFD model to an ex vivo human lung tissue model. These results point toward diet-induced reduction of microbial metabolites, that locally could serve as beneficial signaling molecules during respiratory infections as reported for RSV or RV infection [20, 46, 47].

To further elucidate intracellular processes initiated by acetate at the host-cell level, we conducted experiments on a pulmonary epithelial cell line (A549). Acetate is known to exert its intracellular functions via three main cellular pathways: transporter-mediated entrance. endocytosis, and the engagement of SCFA free-fatty acid receptors, including FFAR2 (GPR43) [48]. To determine whether the observed antiviral effect of acetate was due to impaired viral entry, we utilized pulmonary epithelial cells lacking the SCFA receptor FFAR2 (also known as GPR43). In contrast to wild-type A549 cells, acetate pre-treatment in FFAR2-deficient cells had no impact on viral load, clearly indicating that FFAR2 is essential for mediating the acetate-induced reduction in viral infection (Supplemental Fig. S7). These findings strongly suggest that FFAR2 plays a role in modulating the early stages of infection, likely influencing viral entry. This aligns with the work of Wang et al., who identified FFAR2 as a co-receptor for influenza A virus (IAV) entry during the initial stage of infection [49].

However, in addition to potential effects on virus-cell interaction, FFAR2 engagement is known to initiate intracellular signaling cascades that regulate cellular metabolism and immune responses [50–52]. To further explore these mechanisms, we performed transcriptomic profiling via RNA-sequencing in acetate-treated and IAV-infected A549 cells. This analysis revealed a distinct transcriptional response associated with acetate pre-treatment, including the upregulation of key antiviral genes such as *DDX58* (RIG-I) and *IRF9*, which are central components of the innate immune response and interferon signaling axis [53, 54]. Our data indicate that their expression is enhanced in acetate-treated conditions during IAV infection, suggesting that acetate may potentiate early innate immune recognition of the virus. Moreover, we observed modulation of metabolic pathways, notably those involved in cholesterol metabolism (e.g., *APOE*), glycolysis/gluconeogenesis, and peroxisomal function, suggesting that acetate may exert antiviral activity through both receptor-dependent entry inhibition and broader metabolic reprogramming.

In order to prove the influence of actetate on the interferone signaling, we used IP-10 ko cells. Our results clearly show that IP-10 is essential for the antiviral effect of acetate, as the protective effect is lost in IP-10 knockout cells. IP-10 is a key antiviral effector protein known to directly inhibit viral replication. In the absence of IP-10 in the cell, the virus is no longer effectively controlled, even when acetate is present. This indicates that, in addition to FFAR2 receptor signaling, the induction of a robust antiviral response is crucial for mediating the protective effects of acetate.

Together, these data highlight a dual role of acetate in limiting IAV infection—firstly, through FFAR2-mediated interference with viral entry, and secondly, via downstream transcriptional responses that enhance the host’s antiviral state (Graphical abstract). These mechanisms may be particularly relevant in the context of diet-induced obesity, where systemic levels of microbiota-derived SCFAs such as acetate are reduced, potentially compromising epithelial barrier function and innate immune priming.

## Conclusion

In summary, our study provides compelling evidence that dietary-induced changes in microbial metabolites influence host antiviral responses and Influenza A virus pathogenesis. We showed that HFD exacerbates IAV infection and alters gut microbiota composition, specifically increasing the *Firmicutes/Bacteroidetes* ratio. These changes correspond with reduced expression of genes related to metabolism and carbohydrate-active enzymes. In our DIO mouse model, IAV infection resulted in higher pulmonary viral loads and increased local inflammation. We observed that acetate-producing bacteria and enzymes involved in the synthesis of this key SCFA were reduced in HFD mice, orchestrating the gut-lung-axis. Using the human ex vivo lung model and in vitro experiments with pulmonary epithelial cells, we have shown that acetate pretreatment reduces the viral load through a dual mechanism: by affecting viral entry via the co-receptor FFAR2 and by the dysregulation of intracellular signaling pathways. Notably, acetate increased the expression of antiviral genes such as RIG-1 and IRF9, highlighting its role in modulating the immune response during early IAV infection (Graphical Abstract). These findings suggest that targeting microbial could mitigate viral infections, especially in obese individuals. Further research is needed to clarify the mechanisms by which acetate and other SCFAs modulate immune responses and viral pathogenesis.

## Supplementary Information

Supplementary Material 1: Table S1. Composition of normal-fat diet (NFD) and high-fat diet (HFD). Table S2. Histological scoring system to determine inflammatory status in H&E sections of pulmonary slices from infected mice according to Bergeron et al. [17]. Table S3. Characteristics of serum donors used for analysing SCFA levels. Figure S1. Diagram of metabolizable energy (ME%) of the HFD and control Normal-fat Diet (NFD) from carbohydrates, protein, and fat according to Supplementary Table S1 (A). Representative images of H&E staining of left lung lobes of NFD and HFD mice without (mock) at day 2 and 21 p.i. Scale bar represents 200 µm (B). Qualitative analysis of histoscores based on images as depicted in B, according to Supplemental Tab. S2 at 2 and 21 days post infection (dpi) (C). Figure S2. HFD mice showed altered pulmonary cytokine profiles at acute phase (2 days post infection (dpi), HFD *n*=6, NFD *n*=3) (D) and long-term (21 dpi, HFD *n*=6, NFD *n*=6) (E) compared to corresponding mock mice (NFD, HFD=6). Values are presented as mean ± SD. * *P* <0.05, ** *P* <0.01,*** *P* <0.001, calculated by Kruskal-Wallis test, Dunn’s multiple comparison test. Figure S3. Functional gene annotation during infection and acetate production in NFD and HFD mice. Heatmaps of functional genes annotated to KEGG level 1 and CAZy level 1 and boxplots of acetate synthesis module M00579 in NFD and HFD mice before, 2 days post infection (dpi) and 21 dpi with IAV. Heatmaps are displayed as absolute value of “Z” representing the distance between the raw value and the population mean value in units of the standard deviation (A, B). Acetate levels detected in sera of NFD (*n*=4) and HFD (*n*=4) after 12 weeks of diet (C). Relative abundance of Module 00579 of NFD (*n*=3) and HFD (*n* =3* *P *< 0.05 & *Q<0.05 calculated via Metastats statistical analysis (D). Figure S4. Age and cytokine profile of human serum donor cohorts. Obese healthy female donor cohort (BMI>30, *n*=6) (kg/m² (according to Supplemental Tab. S3) was characterized by increased age compared to non-obese (<25, *n*=4) (A). Cytokines measured in sera from obese and non-obese female donors (B). All values shown in the panels are presented as mean ± SD. ** *P* < 0.01, P calculated by Mann-Whitney test. Figure S5. Acetate does not influence pro-inflammatory lipid mediator secretion in response to IAV infection *ex vivo* but impacts cellular metabolism *in vitro*. Acetate stimulation for 24 h did not increase cytotoxicity in A549 cells as measured by LDH assay (A). PGE2, PGF2a, and TXB2 were increased in supernatants of infected human ex vivo PCLS in response to IAV, independent of acetate pre-treatment, while absent in supernatants of A549 cells. Data are presented as three independent biological replicates per condition (*n*=3) as mean ± SD. (B) Acetate pre-stimulation (*n*=3) altered the differential gene expression (DEG) profile compared to mock (*n* =3) cells at 24 hours post-stimulation (hps), shown in a differential gene volcano map (C). KEGG enrichment analysis revealed altered metabolic processes depicted in a histogram with-log10 adjusted p-value (padj) (D). Heatmaps of differentially expressed genes show increased cholesterol metabolism and glycolysis/gluconeogenesis in infected cells with acetate stimulation (IAVAc) compared to solely infected cells (IAV) at 8 hours post infection (hpi), alongside downregulation of peroxisome-related genes (E). *P <0.05,**P <0.01, **** *P* <0.0001; P calculated by Ordinary one-way ANOVA Tukey’s multiple comparisons test (A, B). Figure S6. Early antiviral effect of acetate during IAV infection in epithelial cells in vitro. Volcano blots displayed acetate pre-treatment with IAV infection (IAVAc) compared with solely infected A549 cells (IAV) and showed altered DEG profiles at 8 hours post infection (hpi) (A). Genes in TLR and TNFα signaling were significantly upregulated in acetate-treated cells compared to unstimulated, infected cells (IAVAcvsIAV) at 8 hpi (B). At 24 hpi, acetate pre-treatment n with IAV infection (IAVAc) led to altered DEG profiles and significant downregulation in Interferon and TNFα signaling genes compared to unstimulated, infected cells (IAV) (C,D). Figure S7. Detailed schematic overview of the proposed mechanism by which acetate stimulation alters GPCR FFAR2 trafficking, resulting in reduced surface expression of FFAR2 as a potential co-receptor for IAV, thereby decreasing viral entry.

## Data Availability

All data supporting this study were made available at Mendeley Data, V1, https://doi.org/10.17632/bbsnbbpx5k.1.

## Abbreviations

AA: Auxiliary Activities
ACSS2: Acetyl-Coa-Synthetase 2
APOE: Apoliporpotein E
BMI: Body mass index
BSA: Bovine serum albumin
CBM: Carbohydrate Binding Molecules
CE: Carbohydrate Esterases
CXCL1: C-X-C Motif Chemokine Ligand 1
DEG: Differentially expressed genes
DIO: Diet-induced obesity
dpi: Days post infection
EMEM: Eagle minimum essential medium
FAR1: Fatty-Acyl-CoA Reducates 1
FBS: Fetal bovine serum
FFAR2: Free fatty acid receptor 2
GH: Glycoside Hydrolases
GM-CSF: Granulocyte-macrophage colony-stimulating factor
GPCR: G protein-coupled recpetor
GPR43: G-protein coupled receptor 43
GT: Glycosyl Transferases
HFD: High-fat diet
hpi: hours post-infection
hps: hours post stimulation
IAV: Influenza A virus
IFIH: Interferon induced with helicase C domain
IFN: Interferon
IL: Interleukin
IP-10: IFN- induced protein (encoded by CXCL10)
IRF: Interferon regulatory factors
ISG: IFN-stimulated genes
KO: Knock out
LDA: Linear discrimant analysis
LefSe: LDA Effect size
MAPK: Mitogen-activated protein kinase
MAVS: mitochondriral antiviral signaling proteins
MCT: Monocarboxylate transporter
MDCK: Madin-Darby canine kidney
MEM: Minimal Essential Medium
ME: Metabolizable energy
MOI: Multiplicity of infection
NFD: Normal-fat diet
NF-B: Nuclear factor kappa-light-chain-enhancer of activated B cells
OAS: Oligoadenylatsynthetase
p.i.: Post infection
padj: adjusted p-value
PCLS: Precision-cut lung slices
PEX11B: Peroxisiomal Biogenesis Factor 11 Beta
PFU: Plaque forming units
PG: Prostaglandine
PI3P: Posphatidylinositol 3-phsopate
PL: Polysaccharide Lyase
PPAR: peroxisome proliferator-activated receptor gamma
PR8: H1N1 Influenza A virus/Puerto Rico/8/34
PRR: Pattern recognition receptor
RANTES: Regulated And Normal T cell Expressed and Secreted
RIG-1: Retinoic acid inducable gene 1
RLR: RIG-1-like receptor
ROS: Reactive oxygen species
RSV: Respiratory syncytial virus
RT: Room temperature
RV: Rhinovirus
SCFA: Short-chain fatty acid
TLR: Toll-like receptor
TNF: Tumor necrosis fcator
TX: Thromboxane
