# Supplementary material for: High-fat diet impairs microbial metabolite production and aggravates influenza A infection
Source: Cell Commun Signal. 2025 Jul 31;23:359. doi: 10.1186/s12964-025-02367-w (PMC12312391; doi:10.1186/s12964-025-02367-w)
Supplement: Supplementary file 1 — Supplementary Material 1: Table S1. Composition of normal-fat diet (NFD) and high-fat diet (HFD). Table S2. Histological scoring system to determine inflammatory status in H&E sections of pulmonary slices from infected mice according to Bergeron et al. [17]. Table S3. Characteristics of serum donors used for analysing SCFA levels. Figure S1. Diagram of metabolizable energy (ME%) of the HFD and control Normal-fat Diet (NFD) from carbohydrates, protein, and fat according to Supplementary Table S1 (A). Representative images of H&E staining of left lung lobes of NFD and HFD mice without (mock) at day 2 and 21 p.i. Scale bar represents 200 µm (B). Qualitative analysis of histoscores based on images as depicted in B, according to Supplemental Tab. S2 at 2 and 21 days post infection (dpi) (C). Figure S2. HFD mice showed altered pulmonary cytokine profiles at acute phase (2 days post infection (dpi), HFD n=6, NFD n=3) (D) and long-term (21 dpi, HFD n=6, NFD n=6) (E) compared to corresponding mock mice (NFD, HFD=6). Values are presented as mean ± SD. * P <0.05, ** P <0.01,*** P <0.001, calculated by Kruskal-Wallis test, Dunn’s multiple comparison test. Figure S3. Functional gene annotation during infection and acetate production in NFD and HFD mice. Heatmaps of functional genes annotated to KEGG level 1 and CAZy level 1 and boxplots of acetate synthesis module M00579 in NFD and HFD mice before, 2 days post infection (dpi) and 21 dpi with IAV. Heatmaps are displayed as absolute value of “Z” representing the distance between the raw value and the population mean value in units of the standard deviation (A, B). Acetate levels detected in sera of NFD (n=4) and HFD (n=4) after 12 weeks of diet (C). Relative abundance of Module 00579 of NFD (n=3) and HFD (n =3* P < 0.05 & *Q<0.05 calculated via Metastats statistical analysis (D). Figure S4. Age and cytokine profile of human serum donor cohorts. Obese healthy female donor cohort (BMI>30, n=6) (kg/m² (according to Supplemental Tab. [file 12964_2025_2367_MOESM1_ESM.docx]

**Supplemental Tables**


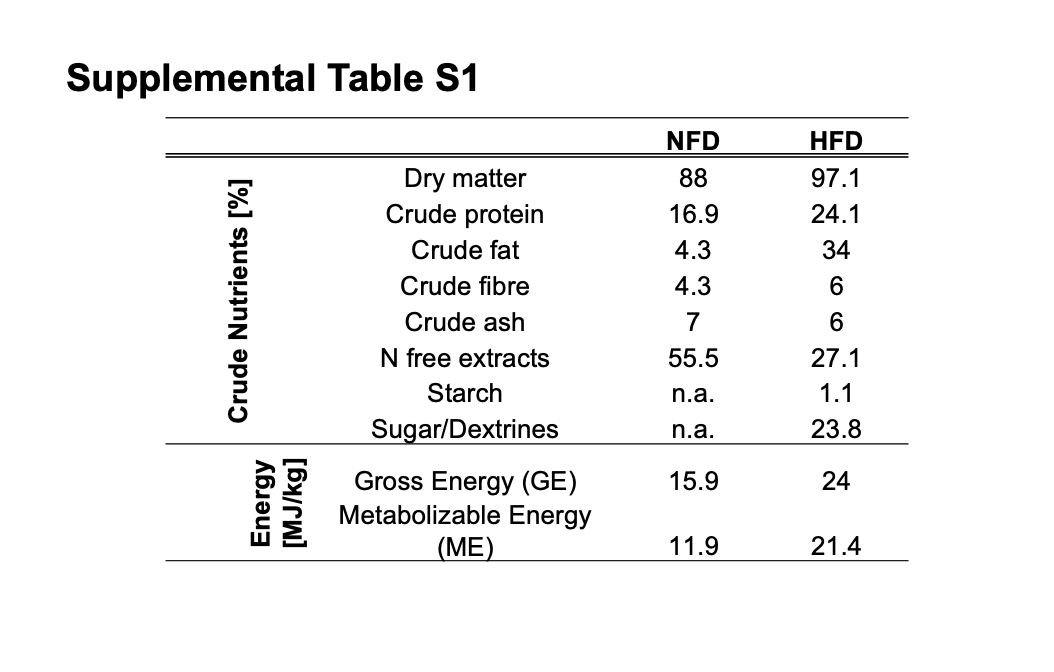


**Supplemental Table S1.** Composition of normal-fat diet (NFD) and high-fat diet (HFD).

**
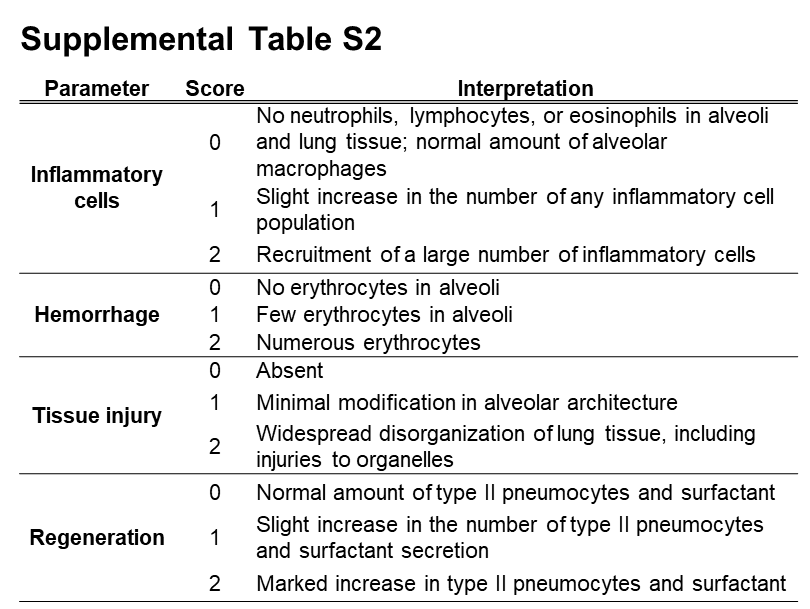
**

**Supplemental Table S2.** Histological scoring system to determine inflammatory status in H&E sections of pulmonary slices from infected mice according to Bergeron et al. (17).

**
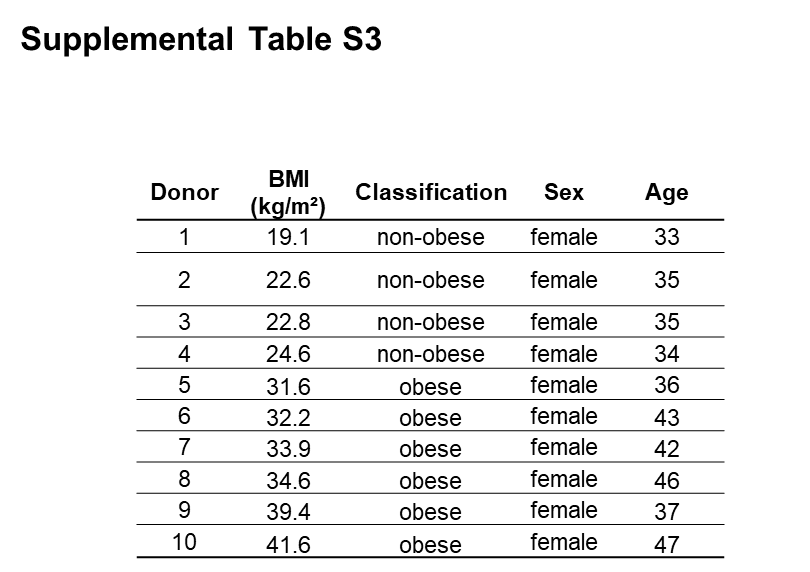
**

**Supplemental Table S3.** Characteristics of serum donors used for analyzing SCFA levels.

**Supplemental Figures**

**
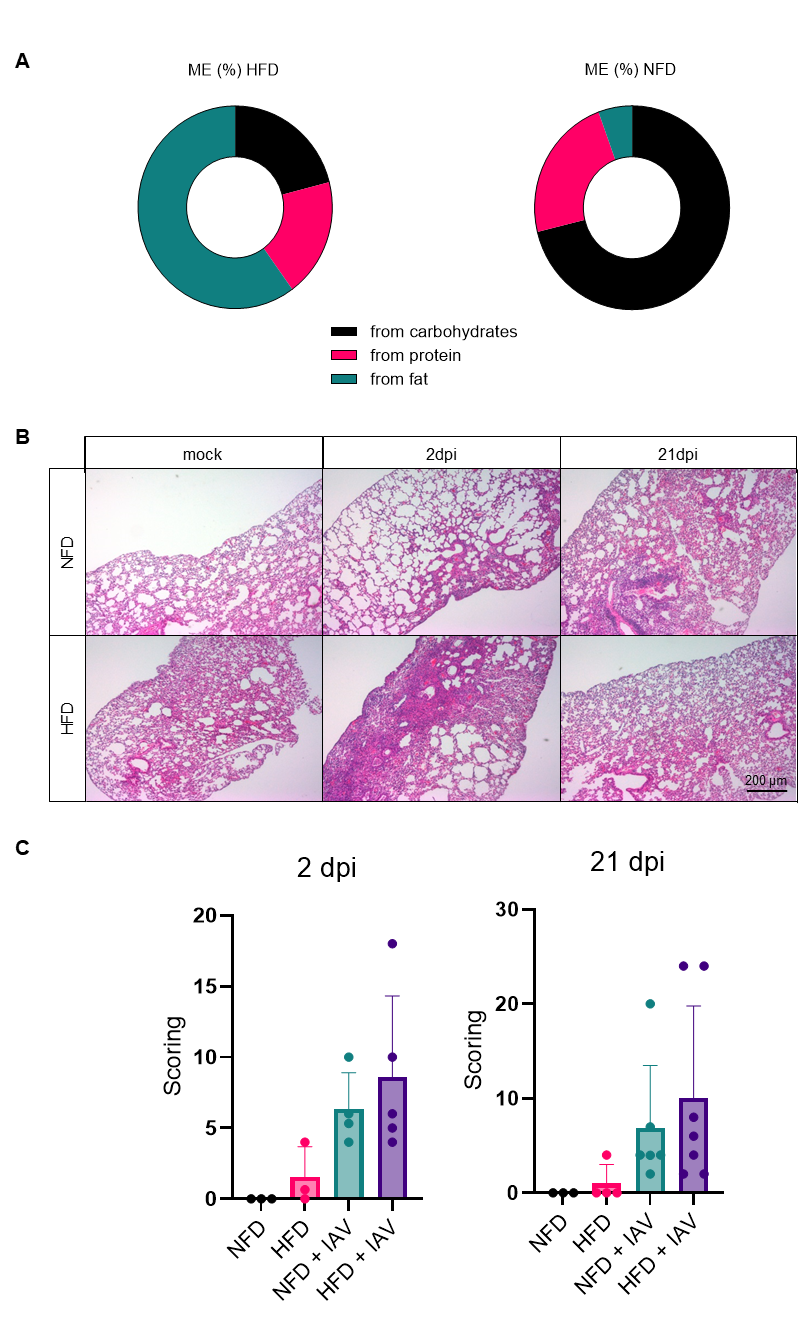
**

**Supplemental Figure S1:** Diagram of metabolizable energy (ME%) of the HFD and control Normal-fat Diet (NFD) from carbohydrates, protein, and fat according to Supplementary Table S1 (A). Representative images of H&E staining of left lung lobes of NFD and HFD mice without (mock) at day 2 and 21 p.i. Scale bar represents 200 µm (B). Qualitative analysis of histoscores based on images as depicted in B, according to Supplemental Tab. S2 at 2- and 21-days post infection (dpi) (C).

**Supplemental Figure S2.** HFD mice showed altered pulmonary cytokine profiles at acute phase (2 days post infection (dpi), HFD n=6, NFD n=3) (D) and long-term (21 dpi, HFD n=6, NFD n=6) (E) compared to corresponding mock mice (NFD, HFD =6) . Values are presented as mean ± SD. *P<0.05, **P<0.01, ***P<0.001, calculated by Kruskal-Wallis test, Dunn’s multiple comparison test.

**
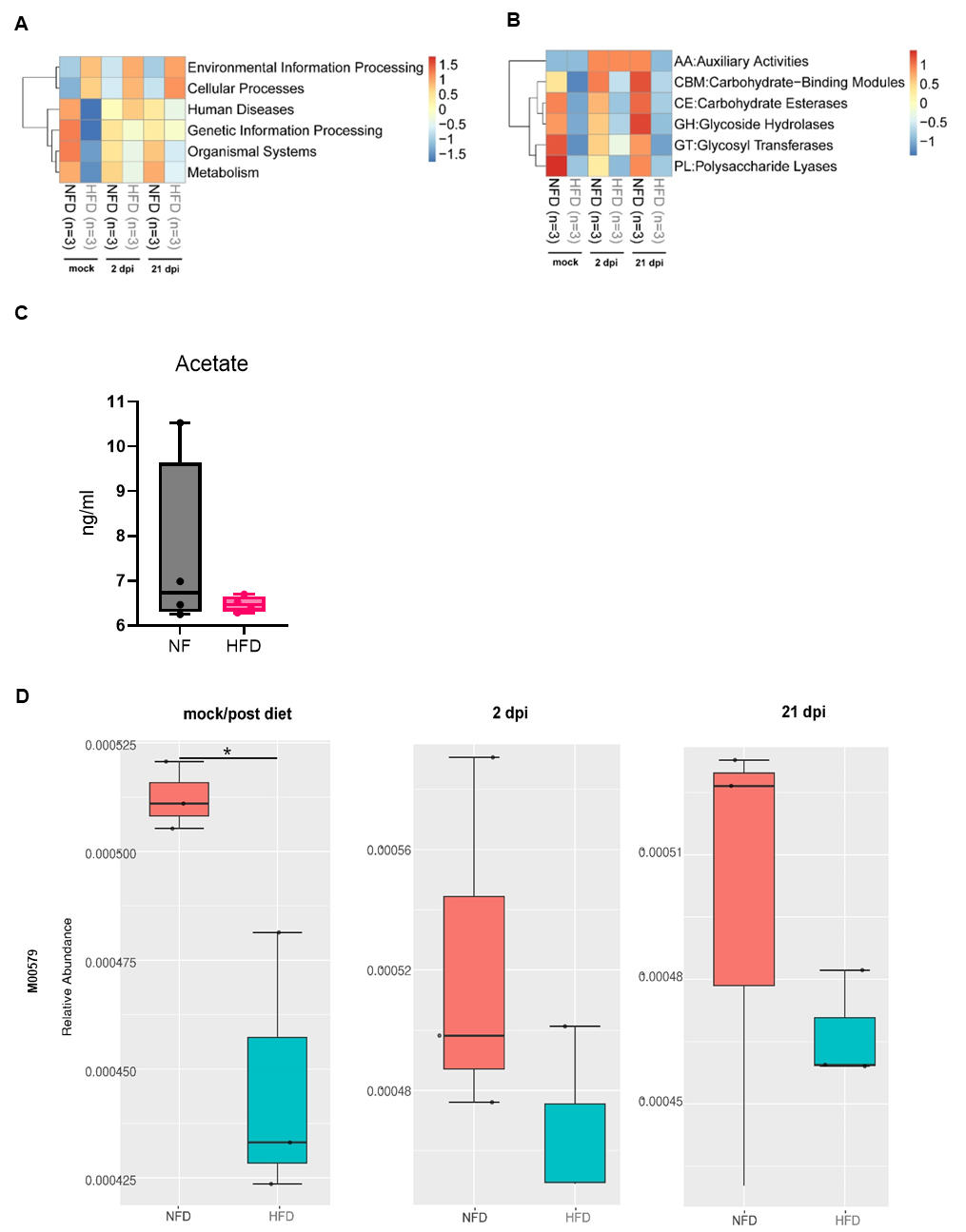
**

**Supplemental Figure S3:** Functional gene annotation during infection and acetate production in NFD and HFD mice. Heatmaps of functional genes annotated to KEGG level 1 and CAZy level 1 and boxplots of acetate synthesis module M00579 in NFD and HFD mice before, 2 days post infection (dpi) and 21 dpi with IAV**.** Heatmaps are displayed as absolute value of “Z” representing the distance between the raw value and the population mean value in units of the standard deviation (A, B). Acetate levels detected in sera of NFD (n=4) and HFD (n=4) after 12 weeks of diet (C). Relative abundance of Module 00579 of NFD (n=3) and HFD (n=3*P<0.05 & *Q<0.05 calculated via Metastats statistical analysis (D).


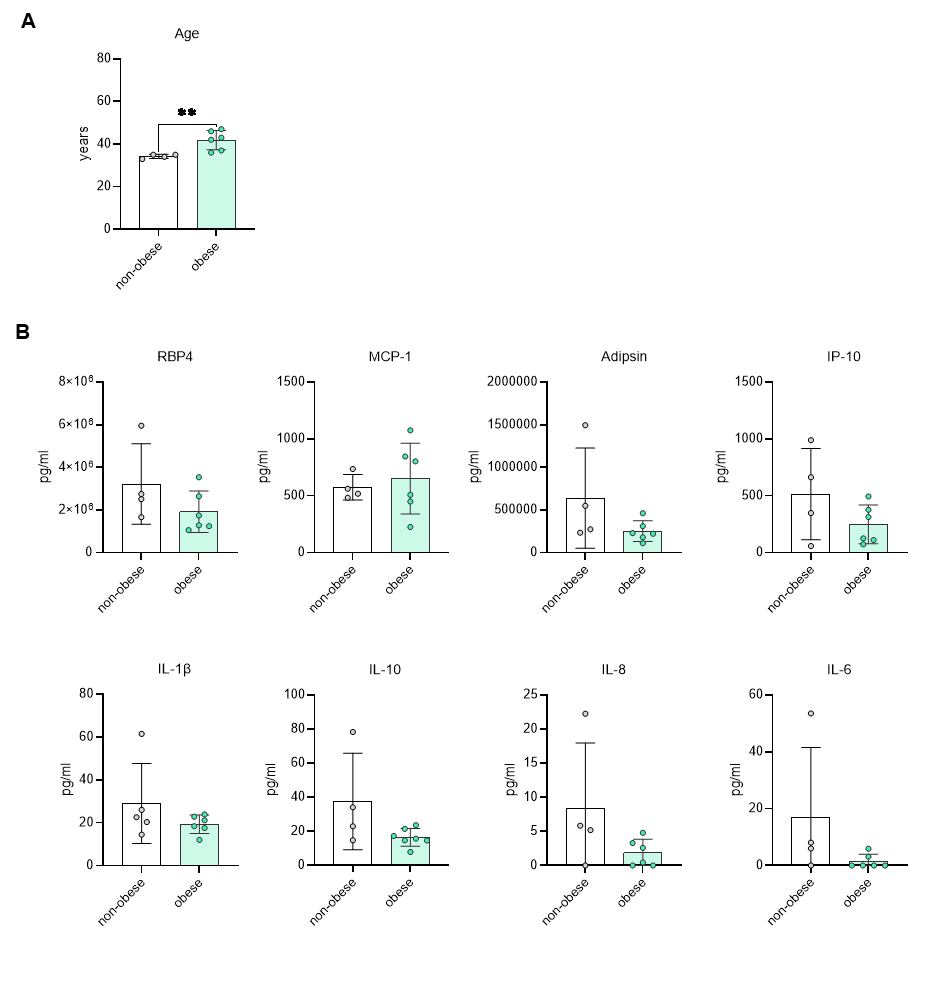


**Supplemental Figure S4. Age and cytokine profile of human serum donor cohorts.** Obese healthy female donor cohort (BMI>30, n=6) (kg/m² (according to Supplemental Tab. S3) was characterized by increased age compared to non-obese (<25, n=4) (A). Cytokines measured in sera from obese and non-obese female donors (B). All values shown in the panels are presented as mean ± SD**.** **P<0.01, P calculated by Mann-Whitney test.


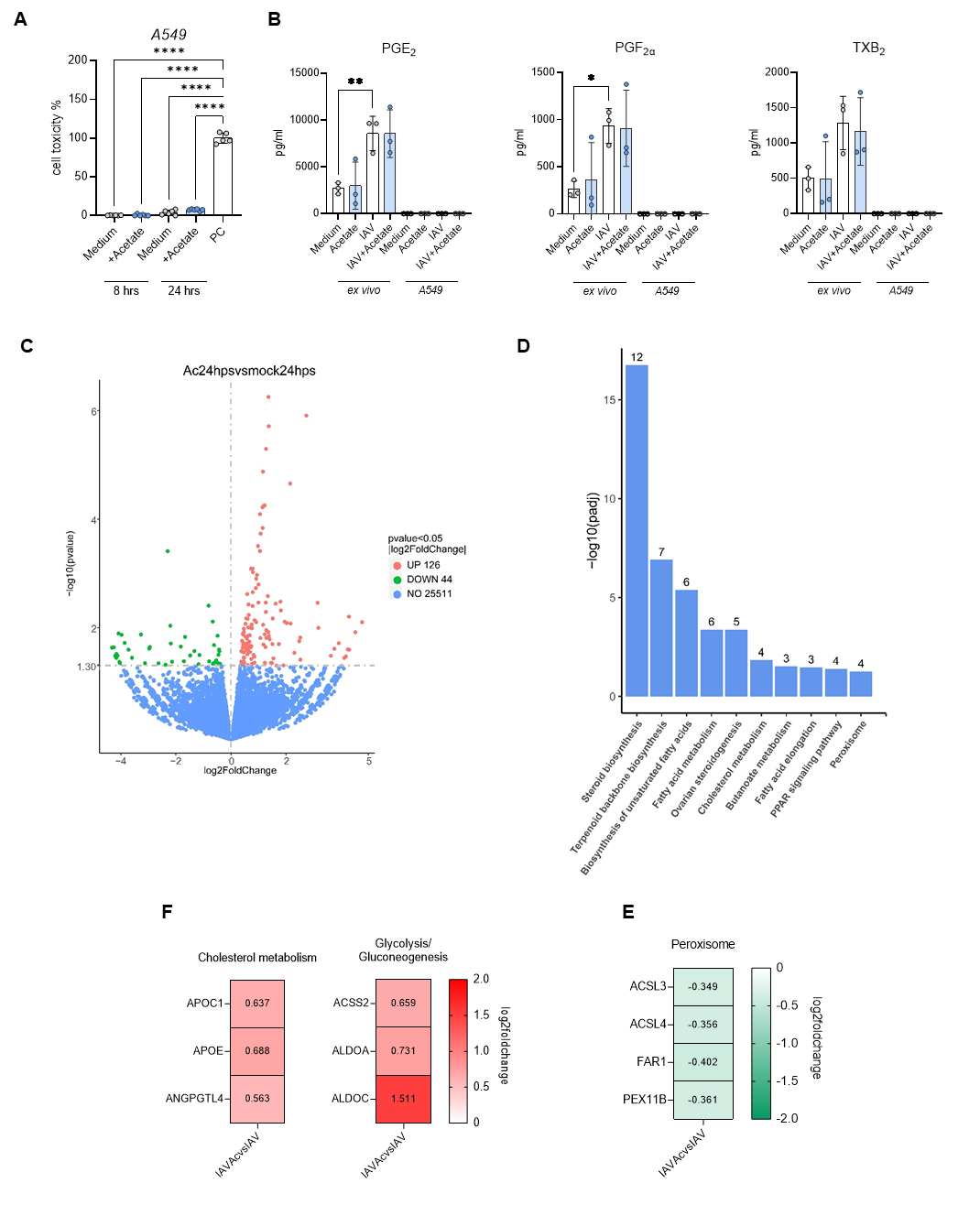
**Supplemental Figure S5.** Acetate does not influence pro-inflammatory lipid mediator secretion in response to IAV infection *ex vivo* but impacts cellular metabolism *in vitro*. Acetate stimulation for 24 h did not increase cytotoxicity in A549 cells as measured by LDH assay (A). PGE2, PGF2a, and TXB2 were increased in supernatants of infected human ex vivo PCLS in response to IAV, independent of acetate pre-treatment, while absent in supernatants of A549 cells. Data are presented as three independent biological replicates per condition (n=3) as mean ± SD. (B) Acetate pre-stimulation (n=3) altered the differential gene expression (DEG) profile compared to mock (n=3) cells at 24 hours post-stimulation (hps), shown in a differential gene volcano map (C). KEGG enrichment analysis revealed altered metabolic processes depicted in a histogram with -log10 adjusted p-value (padj) (D). Heatmaps of differentially expressed genes show increased cholesterol metabolism and glycolysis/gluconeogenesis in infected cells with acetate stimulation (IAVAc) compared to solely infected cells (IAV) at 8 hours post infection (hpi), alongside downregulation of peroxisome-related genes (E). *P<0.05, **P<0.01, **** P<0.0001; P calculated by Ordinary one-way ANOVA Tukey’s multiple comparisons test (A, B).


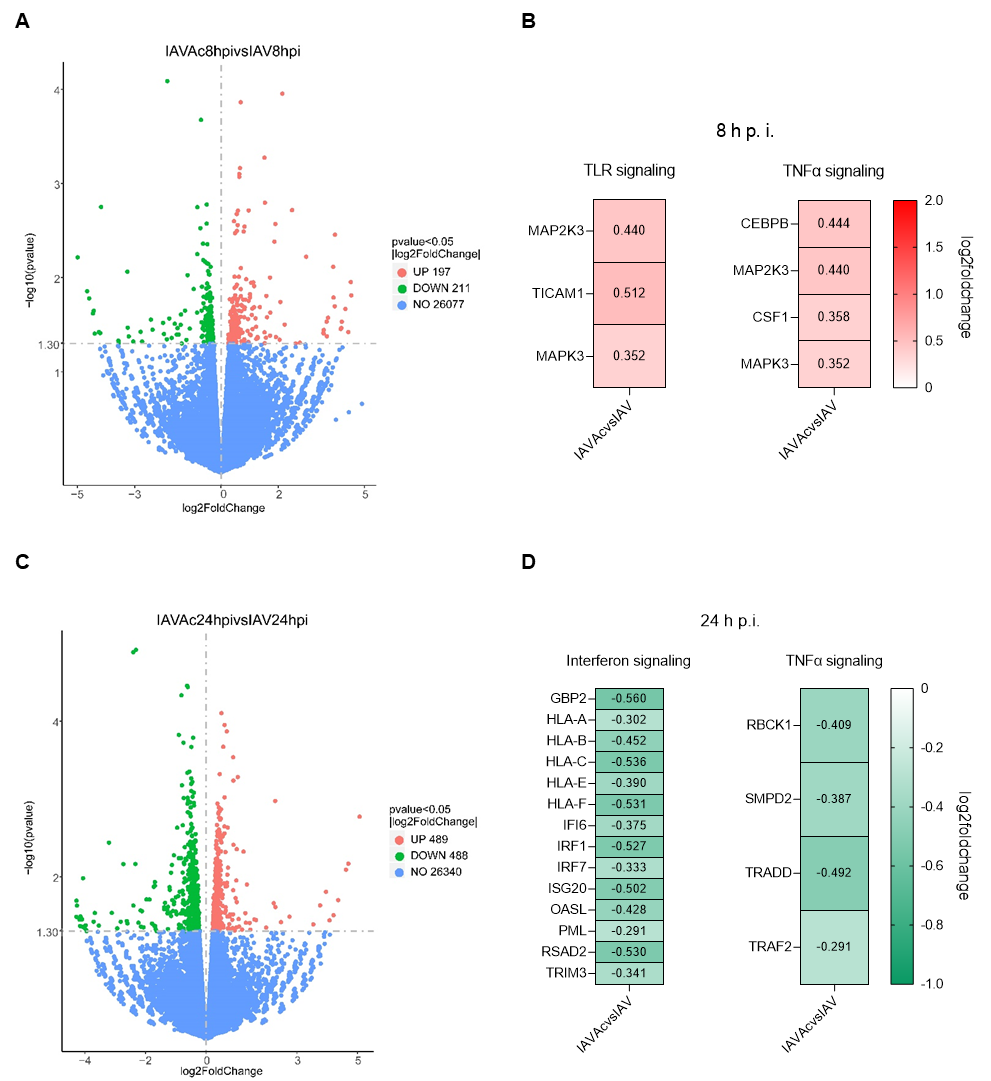


**Supplemental Figure S6. Early antiviral effect of acetate during IAV infection in epithelial cells *in vitro.*** Volcano blots displayed acetate pre-treatment with IAV infection (IAVAc) compared with solely infected A549 cells (IAV) and showed altered DEG profiles at 8 hours post infection (hpi) (A). Genes in TLR and TNFα signaling were significantly upregulated in acetate-treated cells compared to unstimulated, infected cells (IAVAcvsIAV) at 8 hpi (B). At 24 hpi, acetate pre-treatment n with IAV infection (IAVAc) led to altered DEG profiles and significant downregulation in Interferon and TNFα signaling genes compared to unstimulated, infected cells (IAV) (C, D).


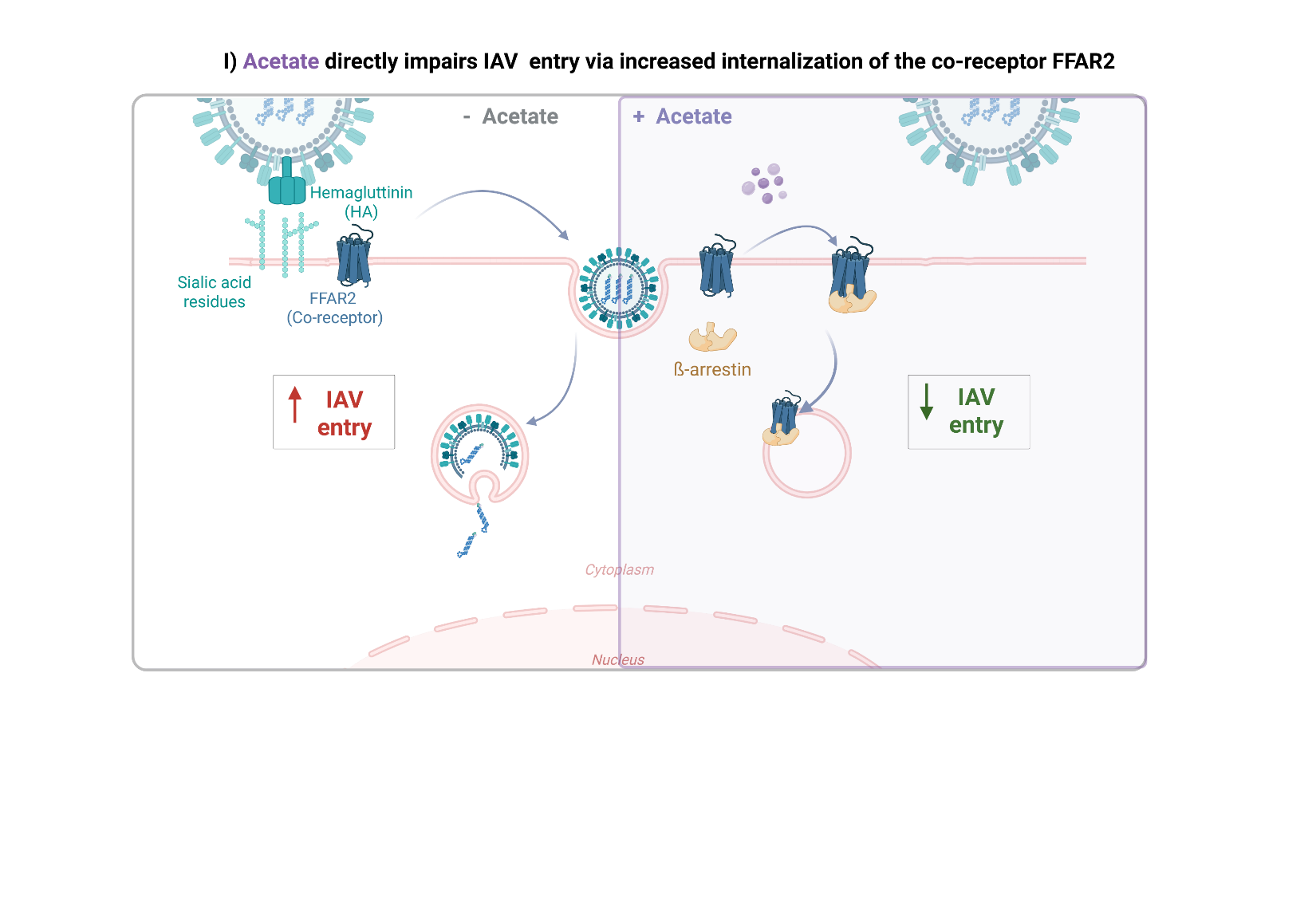


**Supplemental Figure S7**. **Detailed schematic overview of the proposed mechanism by which acetate stimulation alters GPCR FFAR2 trafficking, resulting in reduced surface expression of FFAR2 as a potential co-receptor for IAV, thereby decreasing viral entry.**
